# Adipocyte-mediated epigenomic instability in human T-ALL cells is cytotoxic and phenocopied by epigenetic-modifying drugs

**DOI:** 10.3389/fcell.2022.909557

**Published:** 2022-08-19

**Authors:** Miyoung Lee, Delaney K. Geitgey, Jamie A. G. Hamilton, Jeremy M. Boss, Christopher D. Scharer, Jennifer M. Spangle, Karmella A. Haynes, Curtis J. Henry

**Affiliations:** ^1^ Department of Pediatrics, Emory University School of Medicine and Aflac Cancer and Blood Disorders Center, Children’s Healthcare of Atlanta, Atlanta, GA, United States; ^2^ Department of Microbiology and Immunology, Emory University School of Medicine, Atlanta, GA, United States; ^3^ Winship Cancer Institute, Atlanta, GA, United States; ^4^ Department of Radiation Oncology, Emory University School of Medicine, Atlanta, GA, United States; ^5^ Wallace H. Coulter Department of Biomedical Engineering, Georgia Institute of Technology and Emory University, Atlanta, GA, United States

**Keywords:** obesity, leukemia, epigenetics, cell cycle, genotoxic stress

## Abstract

The world’s population with obesity is reaching pandemic levels. If current trends continue, it is predicted that there will be 1.5 billion people with obesity by 2030. This projection is alarming due to the association of obesity with numerous diseases including cancer, with recent studies demonstrating a positive association with acute myeloid leukemia (AML) and B cell acute lymphoblastic leukemia (B-ALL). Interestingly, several epidemiological studies suggest the converse relationship may exist in patients with T cell acute lymphoblastic leukemia (T-ALL). To determine the relationship between obesity and T-ALL development, we employed the diet-induced obesity (DIO) murine model and cultured human T-ALL cells in adipocyte-conditioned media (ACM), bone marrow stromal cell-conditioned media, stromal conditioned media (SCM), and unconditioned media to determine the functional impact of increased adiposity on leukemia progression. Whereas only 20% of lean mice transplanted with T-ALL cells survived longer than 3 months post-inoculation, 50%–80% of obese mice with leukemia survived over this same period. Furthermore, culturing human T-ALL cells in ACM resulted in increased histone H3 acetylation (K9/K14/K18/K23/K27) and methylation (K4me3 and K27me3) posttranslational modifications (PTMs), which preceded accelerated cell cycle progression, DNA damage, and cell death. Adipocyte-mediated epigenetic changes in human T-ALL cells were recapitulated with the H3K27 demethylase inhibitor GSK-J4 and the pan-HDAC inhibitor vorinostat. These drugs were also highly cytotoxic to human T-ALL cells at low micromolar concentrations. In summary, our data support epidemiological studies demonstrating that adiposity suppresses T-ALL pathogenesis. We present data demonstrating that T-ALL cell death in adipose-rich microenvironments is induced by epigenetic modifications, which are not tolerated by leukemia cells. Similarly, GSK-J4 and vorinostat treatment induced epigenomic instability and cytotoxicity profiles that phenocopied the responses of human T-ALL cells to ACM, which provides additional support for the use of epigenetic modifying drugs as a treatment option for T-ALL.

## Introduction

Among American adults with obesity, defined by body mass index (BMI) greater than 30, the incidence has increased from 30.5% in 1999–2000 to 42.4% in 2017–2018 and is predicted to impact 50% of the population by 2030 (Andolfi and Fisichella 2018). Given that obesity propagates various diseases (Bianchini et al., 2002; Hruby and Hu 2015; Ellulu et al., 2017), this increase has created huge burdens for the health care system (Tsai et al., 2011). A hallmark of obesity is the accumulation of adipocytes, which chronically secrete adipokines and metabolites (Lee and Pratley 2005; Saltiel and Olefsky 2017). Recent studies have demonstrated that these factors promote the growth of cancer cells of varying etiologies directly by providing “fuel” to cancer cells in the form of amino acids (e.g., glutamine) or lipids for β-oxidation. Furthermore, adipocyte-secreted factors can promote tumor growth by attenuating antitumor immunity (Nieman et al., 1999; Marti et al., 2001). The relationship between obesity and solid tumorigenesis is well established; whereas our understanding of how increased adiposity impacts the development of hematological malignancies is still in its earliest stages. Published studies largely demonstrate that mortality rates are higher in persons with obesity diagnosed with B cell acute lymphoblastic leukemia (B-ALL) and acute myeloid leukemia (AML) (Butturini et al., 2007; Ethier et al., 2012; Inaba et al., 2012; Orgel et al., 2014). Despite being controversial (Liu et al., 2021), emerging epidemiological studies suggest that obesity might be protective in cases of T cell acute lymphoblastic leukemia (T-ALL) (Heiblig et al., 2015); however, a mechanistic understanding of how adipocytes impact T-ALL pathogenesis is unknown.

T-ALL, which accounts for roughly 20% of leukemia cases in adult and pediatric populations, is characterized by the rapid proliferation of early lymphoid cells with immature T cell surface markers (Van Vlierberghe and Ferrando 2012; Belver and Ferrando 2016). This cancer is driven by mutations in genes or transcription factors involved in T cell development (Van Vlierberghe and Ferrando 2012; Belver and Ferrando 2016). Although long-term survival of T-ALL can approach 50% in adults (Rowe et al., 2005; Dores et al., 2012) and 90% in pediatric patients (Vora et al., 2013; Place et al., 2015; Moricke et al., 2016), the survival rates of the patients with relapsed or refractory disease are dismal, with a reported 5-year overall survival outcome of less than 30% (Goldberg et al., 2003; Oudot et al., 2008). Unfortunately, our best treatment options have failed to improve survival outcomes in high-risk patients over the past decade, which has prompted the need to define drivers of T-ALL pathogenesis and identify ways to therapeutically exploit novel dependencies.

In cancer, epigenetic modifications mediated by hypermethylation of CpG islands (Esteller 2005), oncohistones (Nacev et al., 2019), IDH1 mutations (Lu et al., 2012), EZH2 expression (Eich et al., 2020), and the SWI/SNF complex (Cenik and Shilatifard 2021) are major drivers of tumorigenesis and disease progression. Sequencing data have revealed that 25% of T-ALL samples analyzed at diagnosis contain genetic lesions in epigenetic modifying enzymes (Ntziachristos et al., 2012; Greenblatt and Nimer 2014; Ntziachristos et al., 2014; Peirs, Van der Meulen et al., 2015). Of these, the polycomb repressor complex 2 (PRC2) is frequently mutated in T-ALL cells (Ntziachristos et al., 2012). This complex is comprised of three protein complexes, EZH2, EED, and SUZ12, which methylate primarily promoter-localized histone H3 at lysine 27 (H3K27), resulting in transcriptional repression (Plath et al., 2003). The methyltransferase activity of this complex is mediated by EZH2, which is responsible for the addition of mono-, di-, and tri-methylation modifications on H3K27. Although EZH2 is overexpressed in many solid cancers, including breast, lung, and liver cancer (Eich et al., 2020), loss-of-function mutations of EZH2, inactivating mutations, or deletion of this epigenetic modifier is commonly present in human T-ALL cells (Ntziachristos et al., 2012; Girardi et al., 2017).

Despite emerging evidence demonstrate that mutations associated with altered epigenomes are common in T-ALL, epigenetic modifying drugs are not traditionally used to treat this disease; whereas, inhibitors targeting histone methyltransferases and histone demethylases are currently being tested in clinical trials as therapies for diffuse large B cell lymphoma (DLBCL) and AML (Morera et al., 2016). In this report, we aimed to determine how obesity impacts T-ALL pathogenesis with an emphasis on defining epigenomic modifications in T-ALL cells, which regulate disease progression. To this end, we found that T-ALL development was suppressed in obese murine models, which supported epidemiological studies reporting the protective effects of obesity in patients with T-ALL (Heiblig et al., 2015). Furthermore, we found that adipocyte-secreted factors directly induced increased transcription and cycle progression with accompanying genotoxic stress and cell death in human T-ALL cells. The increased adipocyte-induced transcription observed in human T-ALL cells was accompanied by alterations in epigenetic states including increases in total H3 protein levels and increased H3 acetylation (K9/K14/K18/K23/K27) and methylation (K4me3 and K27me3). Acetylation of H3 is associated with gene transcription (Di Cerbo et al., 2014), which we observed in our RNA-sequencing studies; however, increased H3 tri-methylation at K4 and K27 is associated with transcriptional activation and repression, respectively (Howe et al., 2017; Guo et al., 2021). Recently, it was noted that H3K4me3 and H3K27me3 also appear at sites of transcription replication conflicts and DNA damage to slow down replication; thus, protecting the genome from DNA damage and instability (Chong et al., 2020). Our results demonstrate that adipocyte-mediated epigenetic changes on H3 are activating, and the increased methylation of H3 at K4 and K27 may mark regions of genomic instability. We also found that the epigenetic modifying drugs GSK-J4 and vorinostat were highly cytotoxic to human T-ALL cells, and epigenetic changes observed in leukemia cells after drug treatment phenocopied changes induced in human T-ALL cells exposed to the adipocyte secretome. Overall, our results demonstrate that adipocytes and epigenetic modifying drugs which increase acetylation and methylation on H3 in human T-ALL cells induce genomic instability and cell death.

## Materials and methods

### Cell lines

T cell acute lymphoblastic leukemia (T-ALL) cells lines were kindly provided from Dr. Christopher Porter and Dr. Douglas Graham laboratories (Department of Pediatrics; Emory University School of Medicine). DND-41, HSB2, Loucy, Molt4, and Peer cells were grown in 20% FBS-supplemented RPMI1640 (Cat#10-041-CV, Corning, NY, United States), and Jurkat cells were grown in 10% FBS-supplemented RPMI1640. The OP-9 mouse bone marrow stromal cell line was grown in 20% FBS-supplemented alpha-MEM (cat#15-012-CV, Corning, NY, United States) and differentiated into adipocytes following previously published protocols (Wolins et al., 2005; Wolins et al., 2006).

### Epigenetic modifying drugs

Inhibitors of epigenetic modifications used in these studies were as follows: GSK-343 (S7164), an EZH2 inhibitor; GSK-J4 (S7070), a H3K27 histone demethylase JMJD3/UTX inhibitor; C646 (S7152), a histone acetyltransferase p300 inhibitor; and vorinostat (SAHA, S1047), a histone deacetylase (HDAC1 and HDAC3) inhibitor. Each drug was purchased from Selleckchem (Houston, TX, United States) and reconstituted in DMSO (cat#D2650, Sigma, St. Louis) as per the manufacturer’s instructions prior to use.

### Cell death assays

Cell death was assessed by using Annexin-V-FITC/PI (cat#BMS500FI-300, ThermoFisher, Waltham, MA, United States) staining, following the manufacturer’s protocol. Briefly, T-ALL cells were conditioned in 10% FBS-supplemented RPMI1640 (control condition), stromal conditioned media (SCM), or adipocyte-conditioned media (ACM) for various time points or treated with epigenetic drugs for 72 h. The cells were harvested, washed with 1X PBS, and stained by Annexin-V-FITC in 1X binding buffer for 15–20 min at room temperature. T-ALL cells were then washed with 1X binding buffers and stained with 10 μg/ml propidium iodide (PI) (J66584, Alfa Aesar, Tewksbury, MA, United States) to assess apoptosis.

### Cell cycle analysis

T-ALL cell cycles were measured using the Click-iT EdU Alexa-Fluor 488 Flow Cytometry Assay Kit (Cat#c10420, ThermoFisher Scientific, Waltham, MA, United States), following the manufacturer’s protocol. Briefly, after conditioning in RPMI, SCM, and ACM or treated with epigenetic drugs, the cells were pulsed with EdU (15 μM) for 2 h at 37°C, followed by fixation in 4% paraformaldehyde in 1X PBS overnight. After washing with 1X PBS, the cells were permeabilized in 1X fixation/permeabilization solution for 15 min at room temperature. T-ALL cells were then stained with anti-EdU-FITC antibody for 30 min at room temperature, followed by PI staining for DNA contents. Results were acquired using a Cytoflex flow cytometer (Beckman Coulter, Indianapolis, United States), and data were analyzed using FlowJo version 10 software (BD, Ashland, United States).

### RNA-sequencing and pathway analysis

Three human T-ALL cell lines (Jurkat, Loucy, and Peer) were cultured for 24 h in RPMI, SCM, or ACM (*n* = 9 samples with one condition/cell line). Total RNA was isolated from leukemia cells using the RNeasy Plus kit (cat#74034, Qiagen, Germantown, United States) and 200–500 ng of total RNA was used as input for the Stranded mRNA-Seq kit with PolyA capture beads (cat# KK8420, KAPA Biosystems) to generate RNA-seq libraries, according to the manufacturer’s instructions. Final libraries were quality checked using a Bioanalyzer (Agilent) and sequenced on a NextSeq500 using PE75 chemistry at the NYU Genome Technology Center.

Analysis of RNA-sequencing data was performed using Reactome, Microsoft Excel, and GraphPad Prism platforms. Normalized data in RPKM uploaded to Reactome and gene expression were analyzed using the camera method. Datasets were annotated according to the cell type (Jurkat, Loucy, and Peer) and media (ACM, SCM, and RPMI); differences between two media types at a time were directly contrasted with cell lines marked as covariates. Output was saved as an expression network diagram that was later cropped to focus on nodes of interest, with the entire diagram included in supplemental data. A total of 23 specific genes of interest for DNA damage and epigenetic modifiers were selected from the global RNA-seq workbook, and the percentage difference in gene expression between media type was calculated for each cell line individually in Excel. Percentage output was added to GraphPad Prism and graphed as percentage change in gene expression from baseline, with a baseline of RPMI media at zero. Raw count expression data were obtained, and count data were normalized and transformed using DESeq2. Genes with no expression across sample groups are filtered out prior to normalization. Furthermore, only genes with greater than zero coverage or read depth in all sample groups were used for normalization and downstream analysis.

### Western blot studies

Human T-ALL cell lines, either treated in conditioned media as previously described or with epigenetic modifiers, were harvested, washed with 1X PBS, and resuspended in 1X RIPA (radio-immunoprecipitation buffer, 150 mM sodium chloride, 50 mM Tris-HCl (pH8.0), 1% NP-40, 0.5% sodium deoxycholate, and 0.1% sodium dodecyl sulfate), containing protease (cat#11836153001) and phosphatase (cat#4906845001) inhibitors (MilliporeSigma, St. Louis, MO, United States) to extract proteins. Protein concentration was quantified using the Pierce BCA protein assay kit (cat#23227, ThermoFisher, Rockford, IL, United States), and 20 μg of protein was analyzed to detect proteins of interest. The protein levels assessed were that of γH2AX (cat#2577s, Cell Signaling Technology, Boston, MA, United States), ERK (p42/44, cat#9102s, Cell Signaling Technology, Boston, MA, United States), pErk [p42/44 (T202/T204), cat#9101s, Cell Signaling Technology, Boston, MA, United States], CHK1 (cat#2360s, Cell Signaling Technology, Boston, MA, United States), pCHK1Ser345 (cat#2348s, Cell Signaling Technology, Boston, MA, United States), CHK2 (cat#6334s, Cell Signaling Technology, Boston, MA, United States), pCHK2Thr68 (cat#2197s, Cell Signaling Technology, Boston, MA, United States), and EZH2 (cat#5246s, Cell Signaling Technology, Boston, MA, United States). For epigenetic assays, we determined the protein levels of EED (cat#PA5-92427, Thermo Fisher Scientific, Waltham, MA, United States), SUZ12 (cat#3737, Cell Signaling Technology, Boston, MA, United States), RING1A (cat#13069s, Cell Signaling Technology, Boston, MA, United States), BMI1 (cat#6964s, Cell Signaling Technology, Boston, MA, United States), H3K27me3 (cat#9733s, Cell Signaling Technology, Boston, MA, United States), H3K4me3 (cat#ab8580, Abcam, Waltham, MA, United States), H3 acetylated on K9/14/18/23/27 (ab47915, Abcam, Waltham, MA, United States), and H3 (cat#9715s, Cell Signaling Technology, Boston, MA, United States). For DNA damage and apoptosis responses, we performed Western blot assays for ATR (cat#2790s, Cell Signaling Technology, Boston, MA, United States), pATR (Ser428) (cat#2853s, Cell Signaling Technology, Boston, MA, United States), p53 (cat#sc-126, Santa Cruz Biotechnology, Dallas, TX, United States), and pp53 (Ser15) (cat#9284s, Cell Signaling Technology, Boston, MA, United States). Either β-actin (cat#4967s, Cell Signaling Technology, Boston, MA, United States) or α-tubulin (cat#2144s, Cell Signaling Technology, Boston, MA, United States) was used as an internal loading control and fluorescent-tagged secondary antibodies, IR Dye 800CW (goat anti-rabbit, cat#926-32211) or 680RD (goat anti-mouse, cat#926–6807, LI-COR Bioscience, Lincoln, United States) were used to detect signals. Signals were visualized using Odyssey (Odyssey CLx, LI-COR Bioscience, Lincoln, United States).

### Histone acetyltransferase and histone deacetylase enzymatic activity assays

Jurkat, Loucy, and Peer T-ALL cells were cultured in RPMI (control), SCM, or ACM for 24 h, after which, leukemia cells were harvested and washed in 1X PBS. Nuclear extracts were prepared, following the manufacturer’s protocol (Cat#OP-0002-1, Epigentek, Farmingdale, NY, United States), and 5 μg was used to measure either HAT (Cat#P-4003, Epigentek, Farmingdale, NY, United States) or HDAC (Cat#P-4034, Epigentek, Farmingdale, NY, United States) enzymatic activity as per the manufacturer’s instructions. Each sample was assayed in duplicate. Enzymatic activities of HAT and HDAC were normalized to responses observed under the RPMI control conditions.

### Chromatin stability assays

To measure the degree of chromatin fragmentation, human T-ALL cell lines were cultured in RPMI, SCM, or ACM for 48 h, and genomic DNA was isolated using DNeasy Blood and Tissue Kit (cat#69504, QIAGEN, Germantown, MD, United States). Total genomic DNA (1 µg) was digested with the Dpn II (cat#R0543, NEB, Ipswich, MA, United States) restriction enzyme, analyzed on 0.8% agarose gel, and imaged using a Gel Doc XR imaging system (Bio-Rad, Hercules, CA, United States) to observe the degree of genomic DNA fragmentation.

To observe abnormal nuclei, we performed immunofluorescence. Briefly, Jurkat cells were plated on 0.01% poly-L-lysine (cat#8920, MiliporeSigma, St. Louis, MO, United States) coated coverslips and cultured in RPMI, SCM, or ACM or treated with GSK-J4 or vorinostat in RPMI for 48 h. The cells were fixed with 4% paraformaldehyde (Cat#J61899, Alfa Aesar, Haverhill, MA, United States) for 15 min at room temperature, followed by three washes with 1X PBS (5 min/wash). The cells were then permeabilized with 0.1% NP-40 in 1X PBS for 15 min at room temperature, washed three times with 1X PBS, followed by blocking for 1 h at room temperature using 10% normal goat serum (Cat#50062Z, Thermo Fisher Scientific, Waltham, MA, United States). The cells were incubated with 1:100 diluted laminin antibody (Cat#Nb300-144ss, Novus Biologicals, Centennial, CO, United States) at 4°C overnight and washed three times with 1X PBS. Alexa-Fluor 568 goat–rabbit IgG (H+L) (1:500 in 10% goat serum, cat#A11011, Invitrogen, Waltham, MA, United States) was used as the secondary antibody and incubated at room temperature for 1 h. T-ALL cells were then washed three times with 1X PBS, followed by one wash with 1X TBS, before mounting with one drop of Prolong Gold Antifade Reagent with DAPI (Cat#p36941, Invitrogen, Waltham, MA, United States). Immunofluorescence images were acquired with the Olympus FV1000 (Center Valley, PA, United States) confocal microscope, and data were analyzed using Fiji–ImageJ software.

### T-ALL murine experiments

Two-month old C57BL/6 mice (The Jackson Laboratory) or NOG mice [Taconic; (genotype) sp/sp;ko/ko (nomenclature) NOD.Cg-*Prkdc* ^*scid*^ *Il2rg* ^*tm1Sug*^/JicTac and (genotype) sp/sp;ko/y (nomenclature) NOD.Cg-*Prkdc* ^*scid*^ *Il2rg* ^*tm1Sug*^/JicTac] were fed control (10% fat calories) or high-fat (60% fat calories) diets for 2–4 months prior to experimentation. The onset of obesity was verified in mice prior to experimentation based on the following criteria: significant weight gain, the chronic production of cytokines/chemokines (IL-6; Invitrogen, cat# 88-7064-22 and TNF-α; Invitrogen, cat# 88-7324-22), and elevated insulin levels (RayBioTech, CODE: ELM-Insulin-1). The chow was purchased from Bio-Serv (cat# F4031 for control diets and cat# S3282 for high-fat diets) and sterilized by irradiation prior to usage. Male and female mice were used for these experiments.

For syngeneic experiments, 2-month old C57BL/6 mice (*n* = 10 mice/diet) were treated with busulfan as previously described as a method of mild myeloablation (Henry et al., 2015), followed by intravenous transplantation with 5 × 10^5^ NOTCH-1-GFP expressing c-kit + cells [created using lentiviral transduction methodology from young (2 month old) hematopoietic stem and progenitor cells as previously described (Henry et al., 2015)]. For xenograft experiments, 10^5^ Peer cells (human T-ALL cell line) were injected intravenously, without conditioning, into NOG mice (*n* = 5 mice/diet). For all experiments, mice were monitored daily for signs of morbidity including hind limb paralysis, labored breathing, abnormal gait, ruffled fur, reduced responsiveness to tactile stimulation, and removed from the study at the first sign of distress per our approved IACUC protocol. Death was not an endpoint for these studies. All murine experiments received ethical approval from the Emory University School of Medicine Institutional Animal Care and Use Committee (IACUC) under the approved protocol number DAR-3000013.

### Real-time PCR gene expression analyses

Human T-ALL cell lines (Jurkat, Loucy, and Peer) were treated in RPMI, SCM, and ACM for 48 h, and RNA was isolated using RNeasy mini kit (Cat#74104, Qiagen, Germantown, MD, United States). Once isolated, 0.8–1 μg of total RNA was used to make cDNA using the Transcriptor First Strand cDNA Synthesis Kit (Cat#04 379 012 001, Roche, Indianapolis, IN, United States). cDNA was diluted 1:5 with H_2_O and 2 μl of diluted cDNA was used to perform real-time PCR using iTaq Universal SYBR Green Supermix (Cat#1725121, Bio-Rad, Hercules, CA, United States). Primers were ordered from Integrated DNA Technologies (IDT, Coralville, IA, United States), and the sequences used for these experiments are listed in Table 1. GAPDH was used as an internal control, and gene expression levels were normalized to expression values found in human T-ALL cells cultured in RPMI.

**TABLE 1:** Real-time PCR primer sequences.

Genes	Sequences	PCR Product Size	References
ATM	Forward: 5′-TGG​ATC​CAG​CTA​TTT​GGT​TTG​A-3′	82bp	PMID: 19661131
	Reverse: 5′-CCA​AGT​ATG​TAA​CCA​ACA​ATA​GAA​GAA​GTA​G-3′		
ATR	Forward: 5′-TGA​AAG​GGC​ATT​CCA​AAG​CG-3′	144bp	PMID: 22319212
	Reverse: 5′-CAA​TAG​ATA​ACG​GCA​GTC​CTG​TCA​C-3′		
CHEK1	Forward: 5′-CAG​GTC​TTT​CCT​TAT​GGG​ATA​CCA​G-3′	122bp	PMID: 22319212
	Reverse: 5′-TGG​GGT​GCC​AAG​TAA​CTG​ACT​ATT​C-3′		
CHEK2	Forward: 5′-AGT​GGT​GGG​GAA​TAA​ACG​CC-3′	117bp	PMID: 28944848
	Reverse: 5′-TCT​GGC​TTT​AAG​TCA​CGG​TGT​A-3′		
CDKN1A	Forward: 5′-CCT​CAT​CCC​GTG​TTC​TCC​TTT-3′	97 bp	PMID: 27572311
	Reverse: 5′-GTA​CCA​CCC​AGC​GGA​CAA​GT-3′		
TP53BP1	Forward: 5′-TGG​CAA​CCC​CGT​GAA​AAT​C-3′	178 bp	PMID: 22319212
	Reverse: 5′-CCA​CCA​CAT​CAA​ATA​CCC​CTA​AAG-3′		
BMI1	Forward: 5′-GGT​ACT​TCA​TTG​ATG​CCA​CAA​CC-3′	124 bp	Origene
	Reverse: 5′-CTG​GTC​TTG​TGA​ACT​TGG​ACA​TC-3′		
EED	Forward: 5′-GAC​GAG​AAC​AGC​AAT​CCA​GAC​C-3′	121 bp	Origene
	Reverse: 5′-TCC​TTC​CAG​GTG​CAT​TTG​GCG​T-3′		
EZH2	Forward: 5′-GAC​CTC​TGT​CTT​ACT​TGT​GGA​GC-3′	115 bp	Origene
	Reverse: 5′-CGT​CAG​ATG​GTG​CCA​GCA​ATA​G-3′		
RING1A	Forward: 5′-CCT​ATC​TGC​CTG​GAC​ATG​CTG​A-3′	127 bp	Origene
	Reverse: 5′-GCT​TCT​TTC​GGC​AGG​TAG​GAC​A-3′		
SUZ12	Forward: 5′-CCA​TGC​AGG​AAA​TGG​AAG​AAT​GTC-3′	135 bp	Origene
	Reverse: 5′-CTG​TCC​AAC​GAA​GAG​TGA​ACT​GC-3′		
GAPDH	Forward: 5′-AGG​GCT​GCT​TTT​AAC​TCT​GGT​AAA-3′	91 bp	PMID: 15153541
	Reverse: 5′-CAT​ATT​GGA​ACA​TGT​AAA​CCA​TGT​AGT​TG-3′		

### Murine and human T cell epigenetic drug treatment assays

Healthy human peripheral blood mononuclear cells (PBMCs; *n* = 3) were a generous gift from Dr. Sunil Raikar’s laboratory (Emory University, Department of Pediatrics, Aflac Cancer and Blood Disorders Center). Naïve CD4^+^ and CD8^+^ T cells were purified using the MiniMACS separator, following the manufacturer’s protocol. Briefly, PBMCs were washed in 1X MACS buffer (2 mM EDTA, pH 8.0, 1% fetal bovine serum in 1X PBS), and the cells were incubated with CD4 (Miltenyi Biotec, Cat#130-045-101) or CD8 (Miltenyi Biotec, Cat#130-045-201) microbeads. T cells were purified using an LS column (Miltenyi Biotec, Cat#130-042-401).

To isolate murine T cells, spleens were harvested from 4-month-old C57BL/6 mice (*n* = 3), and naïve CD4^+^ and CD8^+^ T cells were purified as described earlier using αCD4 (Miltenyi Biotec, Cat#130-117-043) and αCD8 (Miltenyi Biotec, Cat#130-117-044) microbeads, respectively, using MiniMACS magnet separation kit (Cat#130-090-312, Miltenyi Biotech., Gaithersburg, MD, United States).

Human T cells were plated in 10% FBS in RPMI (Cat#10-041-CV, Corning, NY, United States) media supplemented with human IL-7 (50 ng/ml, Cat#200-07, Peprotech, Rocky Hill, NJ, United States) and treated with the epigenetic drugs, GSK-J4, vorinostat, and C646 at concentrations of 25% of the IC_50_, 50% of the IC_50_, and IC_50_ for 72 h. Murine T cells were plated in 10% FBS in RPMI (Cat#10-041-CV, Corning, NY, United States) media supplemented with murine IL-7 (50 ng/ml, Cat#217-17, Peprotech, Rocky Hill, NJ, United States) and treated for 24 h as described previously. In both experiments, the percentage of dead or dying T cells was determined using Annexin-V/PI staining (Cat#BMS500FI-300, Thermo Fisher Scientific, Waltham, MA, United States) followed by flow cytometric analysis. The samples were acquired on the Cytoflex flow cytometer (BD Biosciences), and data were analyzed using FlowJo software (Ashland, Oregon).

### *Ex vivo* human T-ALL cell analysis of DNA damage and apoptosis from lean and obese mice

Lean and obese immunocompromised (NOG) mice were generated as described earlier. Mice fed control or high-fat diet for 2 months were injected intravenously (i.v.) with 10^5^ Peer cells (human T-ALL cell line), without conditioning (*n* = 5 mice/diet). Mice were sacrificed at 20 days post human T-ALL cell injection, prior to the onset of visible signs of morbidity. Spleens were harvested from euthanized mice, and transplanted human T-ALL cells were sorted to greater than 97% purity using αCD45 (Biolegend; Cat#304011) and αCD3 (Biolegend; Cat#300405) antibodies using the Benton Dickison FACS Aria II Cell Sorter. Intracellular staining was performed on purified human T-ALL cells to ascertain the percentage of phospho-γH2AX (Thermo Fisher Scientific; Cat#12-9865-42) and cleaved caspase 3 (Cell Signaling Technology; Cat#12768S) positive T-ALL cells. The samples were acquired using the Cytoflex flow cytometer (BD Biosciences), and data were analyzed using FlowJo software (Ashland, Oregon). All murine experiments received ethical approval from the Emory University School of Medicine Institutional Animal Care and Use Committee (IACUC) under the approved protocol number DAR-3000013.

## Results

### Obesity is protective against T-ALL pathogenesis

Obesity is associated with poor prognosis in multiple solid and hematological cancers (Butturini et al., 2007; Sheng and Mittelman 2014). The tumor promoting property of the obese microenvironment is multifactorial and results in altered pharmacokinetics/pharmacodynamics of chemotherapies (Sheng et al., 2017), compromised cancer immune surveillance (Calle and Kaaks 2004; Iyengar et al., 2016), and the chronic secretion of cytokines, chemokines, and metabolites from adipocytes, which promotes oncogenesis (Park et al., 2014; Jiramongkol and Lam 2020). Although obesity is a well-established risk factor for many cancers, T cell acute lymphoblastic leukemia (T-ALL) is an exception in which, although controversial, significant increases in survival are documented for patients with overweight and obesity (Heiblig et al., 2015; Liu et al., 2021).

**FIGURE 1:**
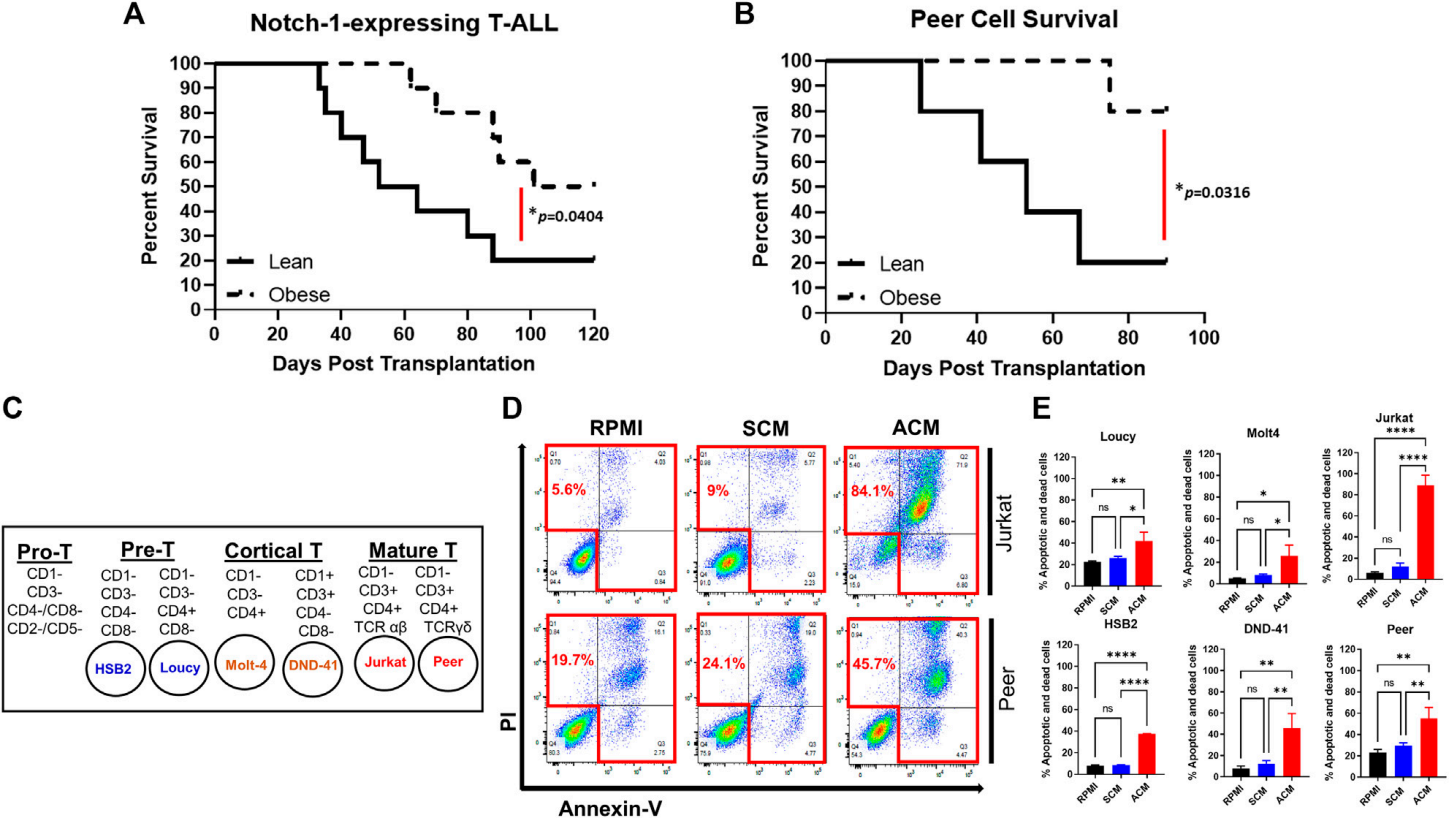
Obesity protects against T-ALL pathogenesis with adipocyte-secreted factors being cytotoxic to human T-ALL cells. **(A)** Murine *c-kit* + cells were isolated from the spleens of C57BL/6 mice and transduced with lentivirus expressing NOTCH1. NOTCH1-expressing *c-kit* cells were injected intravenously *via* tail vein delivery (5 × 10^5^ cells/mouse) into recipient mice (*n* = 10/group) and their survival was monitored. **(B)** Human T-ALL cells (Peer cells) were injected (10^5^ cell/mouse) into lean or obese immunocompromised (NOG) mice as described earlier and survival was monitored. **(C)** List of human T-ALL cell lines used in this study depicting their maturation state as determined by surface immunophenotyping. This figure was adapted from Burger et al. (1999). **(D)** Human T-ALL cells, Jurkat, and Peer, were cultured in RPMI (control condition), stromal cell-conditioned media (SCM), or adipocyte-conditioned media (ACM) for 72 h and cell death was assessed using Annexin-V/PI staining as determined *via* flow cytometric analysis. Primary data from a representative experiment is shown. **(E)** Quantitative data from six independent experiments are shown. Statistical significance in **(A,B)** was determined using a log-rank test and in **(E)** using a one-way ANOVA followed by Tukey’s multiple comparison post-test. *p < 0.05, **p < 0.01, and ****p < 0.0001.

Given the unclear relationship between obesity and T-ALL pathogenesis, we determined how obesity impacted the survival of mice transplanted with murine or human T-ALL cells. For syngeneic experiments, lean and obese C57BL/6 mice were transplanted with hematopoietic stem and progenitor cells (c-kit^*+*^ cells) expressing activated *NOTCH1*, which is a potent driver of T-ALL development found to be mutated in 60% of childhood T-ALL cases (Lee et al., 2005; Garcia-Peydro et al., 2018). Using this approach, we found that while 20% of lean mice transplanted with *NOTCH1*-expressing cells survived over 3 months post-transplantation, this percentage significantly increased to 50% of obese mice over this period (Figure 1A). Similarly, in xenograft experiments where lean and obese immunocompromised mice were transplanted with human T-ALL cells, the survival advantage of obesity was more dramatic with 20% of lean and 80% of obese mice surviving over 2 months post-transplantation with *NOTCH1*-expressing cells (Figure 1B). In xenograft experiments, the increased survival of obese was consistent with increased DNA damage (Supplementary Figure S1A) and caspase 3 activation (Supplementary Figure S1B) observed in transplanted human T-ALL cells at 20 days post-transplantation, prior to signs of morbidity manifesting in mice. These results support epidemiological studies demonstrating superior survival outcomes in obese patients with T-ALL.

### The adipocyte secretome is cytotoxic to human T-ALL cells

To interrogate the mechanism of obesity-mediated suppression of T-ALL pathogenesis, we used a high-throughput *in vitro* system of adiposity to generate large quantities of adipocytes and conditioned media (**CM**) from differentiated bone marrow stromal cells. For these experiments, we determined how the survival of human T-ALL cell lines, derived from both sexes of varying ages, harboring diverse mutations, and presenting at different maturation states (*n* = 6; Table 2; Figure 1C) (Squiban et al., 2017), was impacted after 3 days of culture in unconditioned media, bone marrow stromal cell-conditioned media (**SCM**), and adipocyte-conditioned media (**ACM**). Similar to the obesity-induced suppression of T-ALL pathogenesis *in vivo*, ACM was highly cytotoxic to human T-ALL cells over 3 days of culture (45%–84% cell death) and occurred independently of wild-type or mutant p53 expression (Table 2; Supplementary Figure S2A; Figures 1D,E). In contrast, apoptosis observed in human T-ALL cells cultured in RPMI and SCM was minimal (<25% cell death; Supplementary Figure S2A; Figures 1D,E). A closer analysis of the data revealed that the degree of T-ALL cell death correlated with their differentiation status. The more differentiated human T-ALL cells (Jurkat and Peer T-ALL cell lines) exhibited 45%–84% cell death compared to Loucy [an early T cell progenitor (ETP) cell line] and H2B2 cells, where cell death ranged from 25 to 65% over 3 days of culture in ACM. Despite this observation, cytotoxicity levels in all human T-ALL cells cultured in ACM reached 85%–99%, regardless of the maturation state, with longer (5 day) cultures (Supplementary Figure S2B). In all, these results demonstrate that obesity-induced protection of T-ALL development might result from increased adiposity and the direct cytotoxic effects of the adipocyte secretome on malignant T cells.

**TABLE 2:** Human T-ALL cell line characteristics.

	Origin	NOTCH1	CDKN2A/2B	p53
HSB2	PB of 11 year boy	wt HD and PEST	mut (del)	wt
Loucy	PB of 38 year women	wt HD and PEST	mut (del)	mut (hom, pm)
Molt4	PB of 19 year boy	mut HD (het, pm) PEST (het, del)	mut (hom, del)	mut (het, pm)
DND-41	PB of 13 year boy	mut HD (het, pm) PEST (het, ins/del)	mut (het, del/pm)	mut (hom, pm)
Jurkat	PB of 14 year boy	mut (het, ins)	mut (hom, del)	mut (het, pm)
Peer	PB of 4 year boy	mut (pm)	mut (het, del)	mut (pm)

Wt, wild-type; Mut, mutant; HD, homodomain; PEST, PEST sequence, proline (P), glutamic acid (E), serine (S), and threonine (T); PM, point mutation; Ins, insertion; Del, deletion; Hom, homozygous; Het, heterozygous.

### The adipocyte secretome induces gene expression programs in human T-ALL cells indicative of cell cycle progression, DNA damage, and altered epigenetics

**FIGURE 2:**
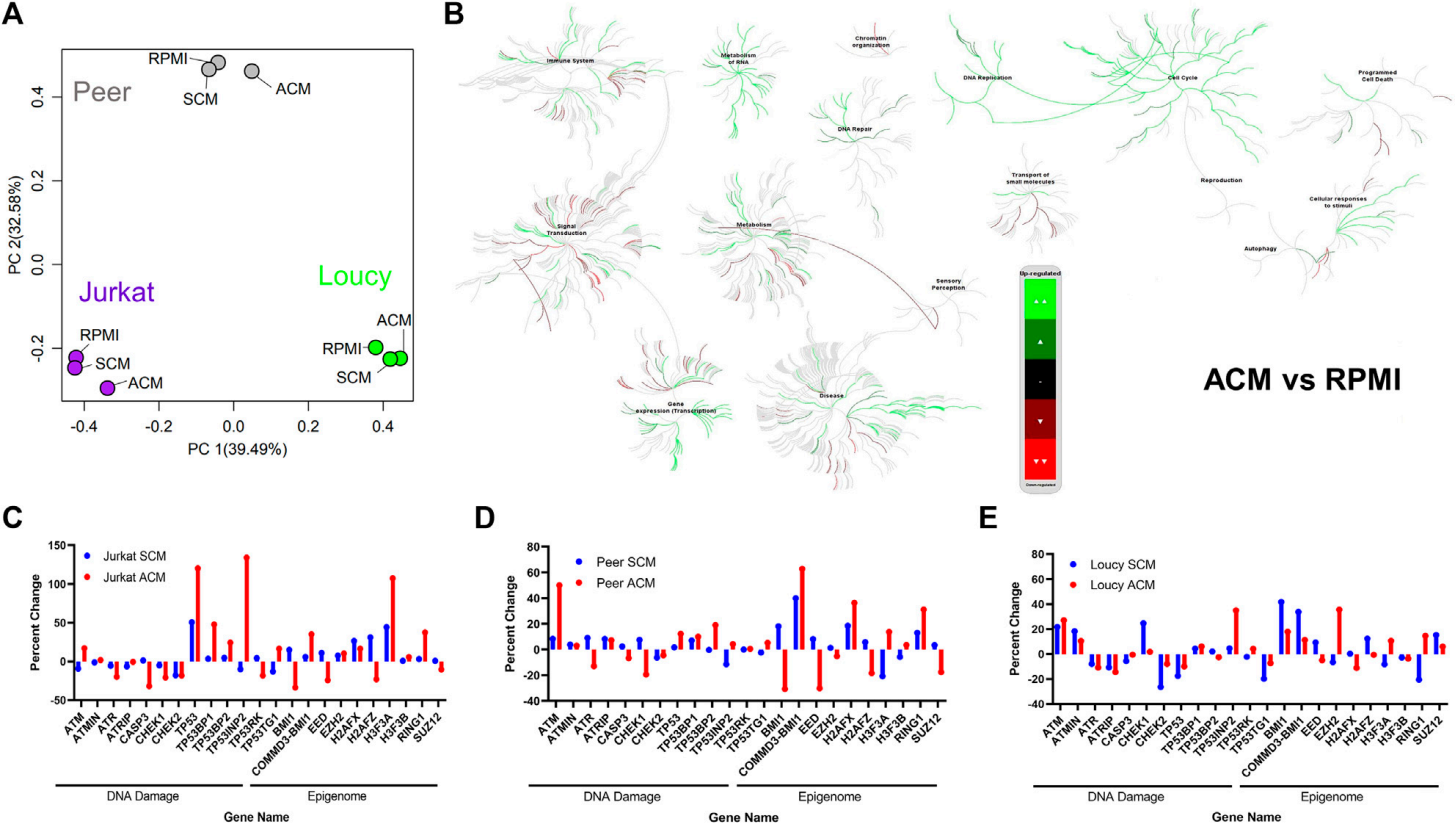
Adipocyte secretome induces gene expression programs in human T-ALL cells indicative of cell cycle progression, DNA damage, and altered epigenetics. RNA-sequencing was performed on human T-ALL cell lines (Jurkat, Loucy, and Peer) after 24 h of culture in unconditioned media RPMI, SCM, and ACM. **(A)** Principal component analysis (PCA) was performed to determine gene expression relationships between leukemia cell lines and culture conditions. **(B)** Data generated from the RNA-sequencing experiments were processed for pathway analysis using Reactome software. Pathways modulated by ACM relative to unconditioned media are shown. **(C–E)** Targeted analysis was performed for genes involved in the DNA damage response (DDR) and epigenetic stability. The fpkm value for each gene in T cells cultured in unconditioned media was used as the control value, and the data are graphed as the percentage increase or decrease relative to control values for each cell line under each condition.

To delineate how the adipocyte secretome modulated gene expression profiles in ACM-exposed human T-ALL cell lines relative to those cultured in unconditioned media and SCM, we performed RNA-sequencing analyses. Principal component analysis (PCA) revealed that a 24-h culture in ACM-induced distinct gene expression changes in the more phenotypically mature Jurkat and Peer T-ALL cell lines; whereas profiles observed in unconditioned and SCM cultured leukemia cells were similar (Figure 2A). In contrast, the ETP ALL cell line, Loucy, exhibited similar gene expression programs when cultured in SCM and ACM relative to unconditioned media, which varied from observations with Jurkat and Peer cells (Figure 2A). In addition to PCA, we performed pathway analysis on the human T-ALL cells cultured under each condition using the Reactome pathway database. To identify pathways commonly up or downregulated in human T-ALL cells cultured in ACM relative to the other conditions tested, gene expression profiles from all three cell lines were combined for each condition. When responses after 24 h of culturing human T-ALL cells in ACM were compared with programs active in leukemia cells cultured in unconditioned media, we observed that ACM upregulated gene expression programs in T-ALL, which were involved in cellular responses to stimuli, cell cycle, DNA replication, DNA repair, metabolism of RNA, and transcription (Figure 2B). Downregulated pathways in ACM-cultured human T-ALL cells include those involved in signal transduction, transport of small molecules, and chromatin organization (Figure 2B). Similar activation and inhibition profiles were observed in ACM-cultured human T-ALL cells relative to SCM-exposed leukemia cells (Supplementary Figure S3). In contrast, culturing human T-ALL cells in SCM relative to unconditioned media did not induce extensive changes in many of the pathways analyzed, with the exception being the modest upregulation of genes involved in the cell cycle, metabolism, and immune response; whereas, those involved in chromatin organization, transcription, and DNA repair were downregulated (Supplementary Figure S4). A list of the most significantly altered pathways in human T-ALL cells cultured in ACM vs. unconditioned media, ACM vs. SCM, and SCM vs. unconditioned media can be found in Tables 3–5, respectively, and increased transcriptional profiles in ACM-stimulated human T-ALL cells was confirmed using quantitative PCR (Supplementary Figures S5A,B).

**TABLE 3:** RNAseq pathway analysis of ACM vs. RPMI (all cell lines combined).

Pathway name	Entities found	*p*-value	FDR	Direction of alteration
Mitochondrial translation	94/102	4.25e-18	1.01e-14	Increased
Mitochondrial translation termination	88/94	1.20e-17	1.18e-14	Increased
Mitochondrial translation elongation	88/94	1.49e-17	1.18e-14	Increased
Mitochondrial translation initiation	88/96	3.92e-17	2.32e-14	Increased
Activation of APC/C and APC/C:Cdc20 mediated degradation of mitotic proteins	77/77	3.20e-16	1.52e-13	Increased
APC/C:Cdc20 mediated degradation of mitotic proteins	76/76	4.28e-16	1.69e-13	Increased
APC:Cdc20 mediated degradation of cell cycle proteins prior to satisfaction of the cell cycle checkpoint	74/74	7.20e-16	2.44e-13	Increased
Regulation of mitotic cell cycle	88/92	1.52e-15	4.01e-13	Increased
APC/C mediated degradation of cell cycle proteins	88/92	1.52e-15	4.01e-13	Increased
Cdc20:Phospho-APC/C mediated degradation of cyclin A	73/73	1.92e-15	4.56e-13	Increased
Regulation of APC/C activators between G1/s and early anaphase	81/83	2.16e-15	4.66e-13	Increased
rRNA modification in the nucleus and cytosol	60/71	7.28e-15	1.44e-12	Increased
Respiratory electron transport, ATP synthesis by chemiosmotic coupling, and heat production by uncoupling proteins	112/153	1.04e-14	1.89e-12	Increased
APC/C:Cdc20 mediated degradation of securin	68/68	1.18e-14	1.99e-12	Increased
APC/C:Cdh1 mediated degradation of Cdc20 and other APC/C:Cdh1 targeted proteins in late mitosis/early G1	74/74	1.52e-14	2.41e-12	Increased
Switching of origins to a post-replicative state	91/93	1.93e-14	2.86e-12	Increased
Synthesis of DNA	120/133	2.15e-14	3.01e-12	Increased
Regulation of ornithine decarboxylase (ODC)	50/51	2.53e-14	3.33e-12	Increased
Autodegradation of Cdh1 by Cdh1:APC/C	64/64	4.78e-14	5.86e-12	Increased
DNA replication	128/142	4.93e-14	5.86e-12	Increased
The citric acid (TCA) cycle and respiratory electron transport	157/235	7.56e-14	8.42e-12	Increased
CDK-mediated phosphorylation and removal of Cdc6	73/75	7.92e-14	8.42e-12	Increased
FBXL7 downregulates AURKA during mitotic entry and in early mitosis	54/55	8.16e-14	8.42e-12	Increased
Vif-mediated degradation of APOBEC3G	55/56	9.19e-14	9.09e-12	Increased
Orc1 removal from chromatin	71/73	1.37e-13	1.30e-11	Increased

**TABLE 4:** RNAseq pathway analysis of ACM vs. SCM (all cell lines combined).

Pathway name	Entities found	*p*-value	FDR	Direction of alteration
Mitochondrial translation	94/102	4.92e-17	1.17e-13	Increased
Mitochondrial translation elongation	88/94	1.11e-16	1.32e-13	Increased
Mitochondrial translation termination	88/94	2.41e-16	1.91e-13	Increased
Mitochondrial translation initiation	88/96	4.25e-16	2.53e-13	Increased
rRNA modification in the nucleus and cytosol	60/71	7.55e-15	3.59e-12	Increased
mRNA splicing—major pathway	177/185	5.10e-12	2.02e-09	Increased
mRNA splicing	185/196	8.92e-12	2.74e-09	Increased
Processing of capped intron-containing pre-mRNA	240/256	9.24e-12	2.74e-09	Increased
Synthesis of DNA	120/133	3.41e-11	8.29e-09	Increased
DNA replication	128/142	3.49e-11	8.29e-09	Increased
Switching of origins to a post-replicative sate	91/93	1.24e-10	2.67e-08	Increased
DNA replication pre-initiation	85/88	1.71e-10	3.25e-08	Increased
Respiratory electron transport, ATP synthesis by chemiosmotic coupling, and heat production by uncoupling proteins	112/153	1.78e019	3.25e-08	Increased
APC:Cdc20 mediated degradation of cell cycle proteins prior to satisfaction of the cell cycle checkpoint	74/74	2.00e-10	3.39e-08	Increased
Activation of APC/C and APC/C:Cdc20 mediated degradation of mitotic proteins	77/77	2.92e-10	4.63e-08	Increased
Regulation of mitotic cell cycle	88/92	3.33e-10	4.66e-08	Increased
APC/C mediated degradation of cell cycle proteins	88/92	3.33e-10	4.66e-08	Increased
S phase	162/180	4.29e-10	5.16e-08	Increased
APC/C:Cdc20 mediated degradation of mitotic proteins	76/76	4.42e-10	5.16e-08	Increased
Cdc20:Phospho-APC/C mediated degradation of Cyclin A	73/73	4.45e-10	5.16e-08	Increased
Orc1 removal from chromatin	71/73	4.56e-10	5.16e-08	Increased
G1/S transition	145/150	4.99e-10	5.21e-08	Increased
Regulation of APC/C activators between G1/S and early anaphase	81/83	5.04e-10	5.21e-08	Increased
Eukaryotic translation elongation	94/102	1.03e-09	1.02e-07	Decreased
The citric acid (TCA) cycle and respiratory electron transport	157/235	1.08e-09	1.03e-07	Increased

**TABLE 5:** RNAseq pathway analysis of SCM vs. RPMI (All cell lines combined).

Pathway name	Entities found	*p*-value	FDR	Direction of alteration
Eukaryotic translation elongation	94/102	4.14e-20	7.10e-17	Increased
Peptide chain elongation	90/97	5.97e-20	7.10e-17	Increased
Viral mRNA translation	91/114	2.15e-19	1.71e-16	Increased
Eukaryotic translation termination	94/106	2.34e-18	1.39e-15	Increased
Nonsense mediated decay (NMD) independent of the exon junction complex (EJC)	96/101	6.77e-18	3.22e-15	Increased
Formation of a pool of free 40S subunits	102/106	1.63e-17	6.46e-15	Increased
Selenocysteine synthesis	94/112	9.87e-17	3.35e-14	Increased
L13a-mediated translational silencing of ceruloplasmin expression	112/120	3.14e-16	9.33e-14	Increased
GTP hydrolysis and joining of the 60S ribosomal subunit	113/120	1.54e-15	4.08e-13	Increased
Eukaryotic translation initiation	120/130	2.58e-14	5.57e-12	Increased
Cap-dependent translation initiation	120/130	2.58e-14	5.57e-12	Increased
SRP-dependent cotranslational protein targeting to membrane	113/119	3.67e-14	7.27e-12	Increased
Response of EIF2AK4 (GCN2) to amino acid deficiency	105/115	2.78e-13	5.08e-11	Increased
Regulation of expression of SLITs and ROBOs	164/183	6.17e-13	1.05e-10	Increased
Nonsense mediated decay (NMD)	117/124	2.11e-12	3.13e-10	Increased
Nonsense mediated decay (NMD) enhanced by the exon junction complex (EJC)	117/124	2.11e-12	3.13e-10	Increased
Influenza viral RNA transcription and replication	141/176	2.27e-12	3.18e-10	Increased
Selenoamino acid metabolism	113/180	3.92e-11	5.17e-09	Increased
Influenza infection	160/200	1.01e-09	1.26e-07	Increased
Signaling by ROBO receptors	202/235	1.08e-09	1.29e-07	Increased
Translation initiation complex formation	59/62	3.39e-09	3.84e-07	Increased
Formation of the ternary complex, and subsequently, the 43S complex	52/54	4.36e-09	4.71e-07	Increased
Ribosomal scanning and start codon recognition	59/64	1.24e-08	1.25e-06	Increased
Activation of the mRNA upon binding of the cap-binding complex and eIFs, and subsequent binding to 43S	60/66	1.26e-08	1.25e-06	Increased
Metabolism of amino acids and derivatives	297/661	5.45e-06	5.18e-04	Increased

Given the extensive increase in DNA replication and transcription with accompanying decreases in chromatin organization pathways in human T-ALL cells exposed to the ACM relative to SCM or unconditioned media, we mined our RNA-sequencing data to query the expression of genes, which regulate DNA repair, cell cycle progression, apoptosis, and epigenetic states. The gene expression levels observed in human T-ALL cells cultured in RPMI were used as the baseline (0% level). In support of the induction of DNA damage programs in human T-ALL cells cultured in ACM, we observed increased gene expression levels of *ATM*, which responds primarily to double-stranded breaks (DSBs), in leukemia cells exposed to the adipocyte secretome; whereas, this gene was downregulated in T-ALL cells cultured in SCM (with the exception of Loucy cells; Figures 2C–E). Furthermore, the gene encoding ATR, which is serine/threonine-specific kinase involved in sensing DNA damage and activating the DNA damage checkpoint, was more extensively suppressed in ACM-cultured human T-ALL cells (Figures 2C–E). In support of the hypothesis that human T-ALL cells cultured in ACM may not effectively activate cell cycle checkpoints when experiencing DNA damage, *CHEK1* and *CHEK2* gene expressions were lower in human T-ALL cells when cultured in ACM relative to RPMI (and in some cases, SCM), with responses being more apparent in the more differentiated human T-ALL cells (Jurkat and Peer; Figures 2C–E). In addition, genes coding for TP53, or its binding partners, were largely upregulated in human T-ALL cells cultured in ACM relative to the other conditions tested (Figures 2C–E). Notable changes in genes regulating the epigenome were also observed. The gene expression levels of *BMI1*, which is rapidly recruited to sites of double-stranded DNA breaks (Ismail et al., 2010), were extensively downregulated in more differentiated human T-ALL cells cultured in ACM; whereas, the converse was true in the Loucy ETP ALL cell line (Figures 2C–E). Furthermore, the gene encoding the polycomb protein EED, which is involved in maintaining the transcriptional repressive state of genes (Obier et al., 2015), was suppressed in all human T-ALL cell lines tested when cultured in ACM relative to the other conditions tested; whereas, *RING1*, which is also part of the polycomb complex (Stock et al., 2007), was upregulated when human T-ALL cells were exposed to the adipocyte secretome (Figures 2C–E). In addition to ACM-mediated changes in gene expression regulators in human T-ALL cells, genes coding for histones were drastically altered in human T-ALL cultured in ACM relative to the other conditions tested (Figures 2C–E). Specifically, the *H2AFZ* gene (which encodes H2A.Z.1) was downregulated in ACM-cultured T-ALL cells (Figures 2C–E), and high expression levels of this gene are associated with more aggressive solid cancers such as hepatocellular carcinoma (Dong et al., 2021). In contrast, the *H3F3A* gene (which encodes H3.3) was upregulated in ACM-exposed T-ALL cells (Figures 2C–E). This observation is consistent with its role in increasing transcription (Park et al., 2016) and our pathway analysis results of human T-ALL cells cultured in the adipocyte secretome (Figure 2B; Supplementary Figure S3). Furthermore, H3.3 histones are deposited after DNA damage (Pinto et al., 2021), and the upregulation of *H3F3A* may result from the increased genomic instability observed in human T-ALL cells cultured in ACM (Figure 2B; Supplementary Figure S3). In addition, we observed that the gene expression levels of *H2AFX* were variable among ACM-cultured human T-ALL cells; whereas the *H3F3B* gene was expressed at similar levels under all conditions tested. This analysis identified adipocyte-induced transcriptional changes in human T-ALL cells, which precede cell death (Figure 1; Supplementary Figure S2).

### The adipocyte secretome induces cell cycle progression in human T-ALL cells with concomitant DNA damage

**FIGURE 3:**
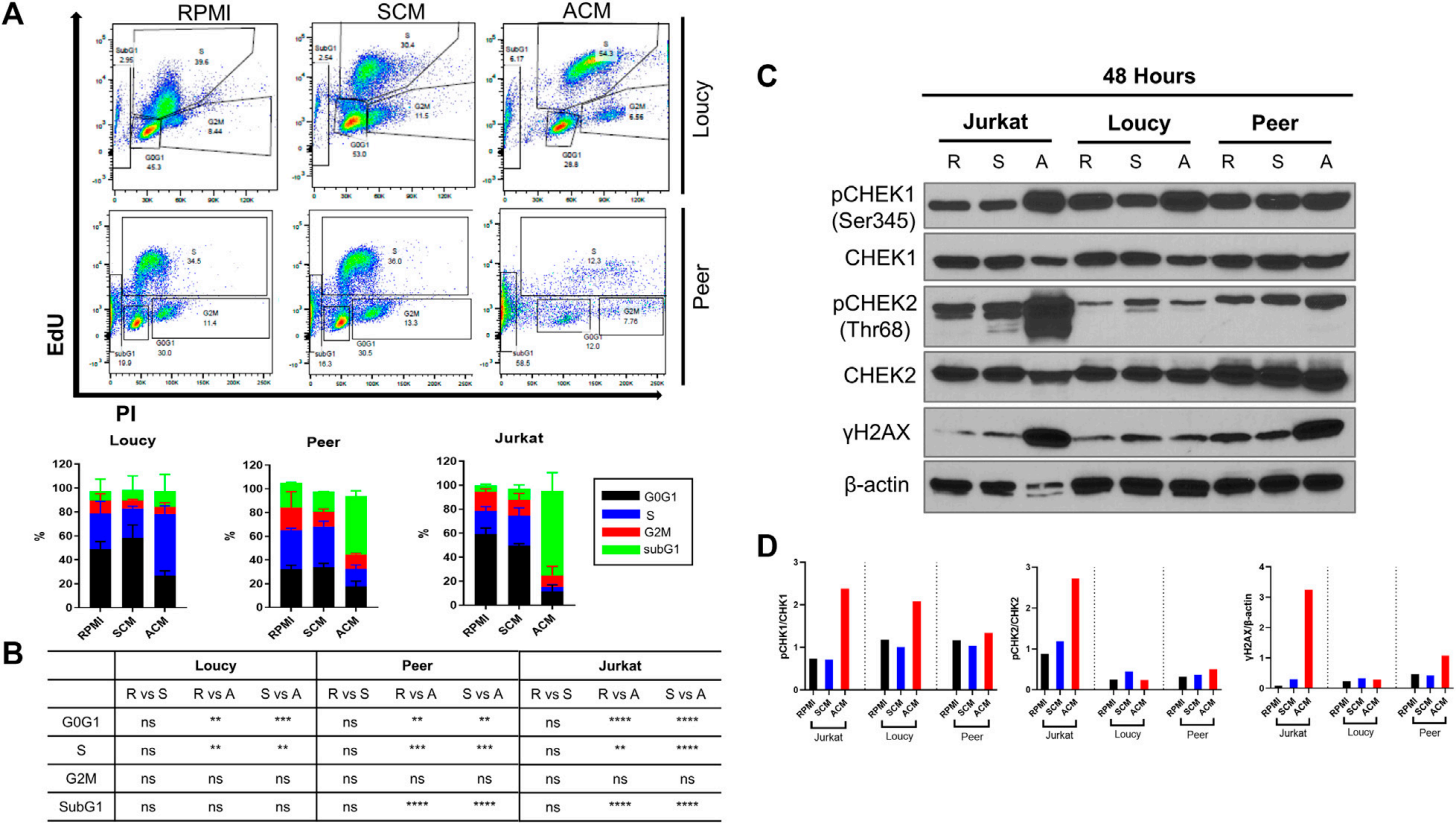
Adipocyte secretome induces cell progression in human T-ALL cells with concomitant DNA damage. **(A)** Human T-ALL cells (Loucy and Peer) were cultured in unconditioned media RPMI, SCM, or ACM for 72 h, and cell cycle progression was determined using EdU analysis *via* flow cytometry. Representative flow cytometry data are shown. **(B)** Quantitative results are shown for three independent experiments. **(C)** Human T-ALL cells (Jurkat, Loucy, and Peer) were cultured for 48 h as described in **(A),** and Western blot analysis was performed to detect the protein levels of the indicated cell cycle checkpoint regulators (CHEK1/CHEK2) and indicators of genotoxic stress (γH2AX). Primary data from a representative experiment is shown. **(D)** Protein expression levels from the Western blot experiments were determined using ImageJ analysis and a representative blot from two independent experiments is shown. R = RPMI, S = SCM, and A = ACM in **(B,C)**.

Based on our RNA-sequencing analysis, which revealed substantial alterations in genes regulating the cell cycle and checkpoint responses to double-strand DNA breaks (*ATM*, *ATR*, *CHEK1*, and *CHEK2*) when human T-ALL cells were exposed to the adipocyte secretome, we next assessed cell cycle progression in leukemia cells cultured in unconditioned media, SCM, and ACM. After 3 days of culture, every human T-ALL cell line cultured in ACM exhibited increased cell cycle progression relative to responses observed in leukemia cells cultured in unconditioned or stromal cell-conditioned media (Supplementary Figure S6A; Figures 3A,B). Notably, the percentage of leukemia cells in the SubG1 population was higher in more differentiated human T-ALL cells (Jurkat and Peer) cultured in ACM (Figures 3A,B), which is indicative of increased cell death (Supplementary Figure S2; Figure 1). Similar to the slower apoptotic responses observed in the ETP ALL cell line (Loucy), a higher percentage of cells were observed in S phase, compared to SubG1, when cultured in ACM relative to the other conditions tested (Figures 3A,B).

Given that cell cycle progression was induced in every human T-ALL cell line cultured in ACM, and our gene expression profiles demonstrating adipocyte-induced alterations in *CHEK1* and *CHEK2* levels in malignant T cells, we next determined total and activated CHEK1 and CHEK2 protein levels in leukemia cells cultured under each condition. The protein levels of CHEK1 were similar in all human T-ALL cell lines tested after 24 h of culture in unconditioned or conditioned media (Supplementary Figure S4B); whereas, paralleling the reduced gene expression levels observed, there were lower CHEK1 protein levels in Jurkat and Loucy T-ALL cells cultured for 48 h in ACM (Figure 3C). Unlike CHEK1 and CHEK2 total protein levels were equivalent in all T-ALL cells cultured in unconditioned and conditioned media at 24 and 48 h (Supplementary Figure S6B; Figure 3C). In support of ACM-induced DNA damage altering the cell cycle of human T-ALL cells, we observed rapid (Jurkat cells at 24 h) and increased (Jurkat and Peer cells at 48 h) γH2AX protein expression in human T-ALL cells cultured in ACM relative to the other conditions tested (Supplementary Figure S6B; Figures 3C,D). This response occurred concomitantly with increased activation of CHEK1 and to a lesser extent CHEK2 (Supplementary Figure S6B; Figures 3C,D). These observations suggested that malignant T-ALL cells were unsuccessfully attempting to repair damaged DNA due to increased cell cycle progression when cultured in ACM. In support of this hypothesis, we observed decreased activation of ATR (reduced pATR protein levels) in all human T-ALL cell lines cultured in ACM for 48 h relative to the other conditions tested while total protein levels remained equivalent (Supplementary Figure S6C). The significant increase in ACM-induced apoptosis observed in human T-ALL cells (Supplementary Figure S2; Figure 1) could not be explained by altered p53 protein levels or increased p53 activation, which could potentially be attributed to the mutated status of this tumor suppressor in the cell lines tested (Table 2). The DNA damage response (DDR) is activated in cells accumulating single-stranded breaks/replication stress or double-stranded breaks with the goal of repairing DNA lesions (Rouse and Jackson 2002; Harrison and Haber 2006; Harper and Elledge 2007). The DDR is mediated by ATR/CHEK1 (Cimprich and Cortez 2008) for single-stranded breaks or ATM/CHEK2 (Shiloh 2003) for double-stranded breaks, which results in DNA repair, cell cycle arrest, or apoptosis if lesions are not repaired as the cell cycle progresses (Jackson and Bartek 2009). Collectively, these results demonstrate that the ACM-induced T-ALL cell death is preceded by deregulation of the cell cycle with accompanying DNA lesions, which occurs concurrently with reductions in phospho-ATR activation.

### The adipocyte secretome alters the epigenome of human T-ALL cells

**FIGURE 4:**
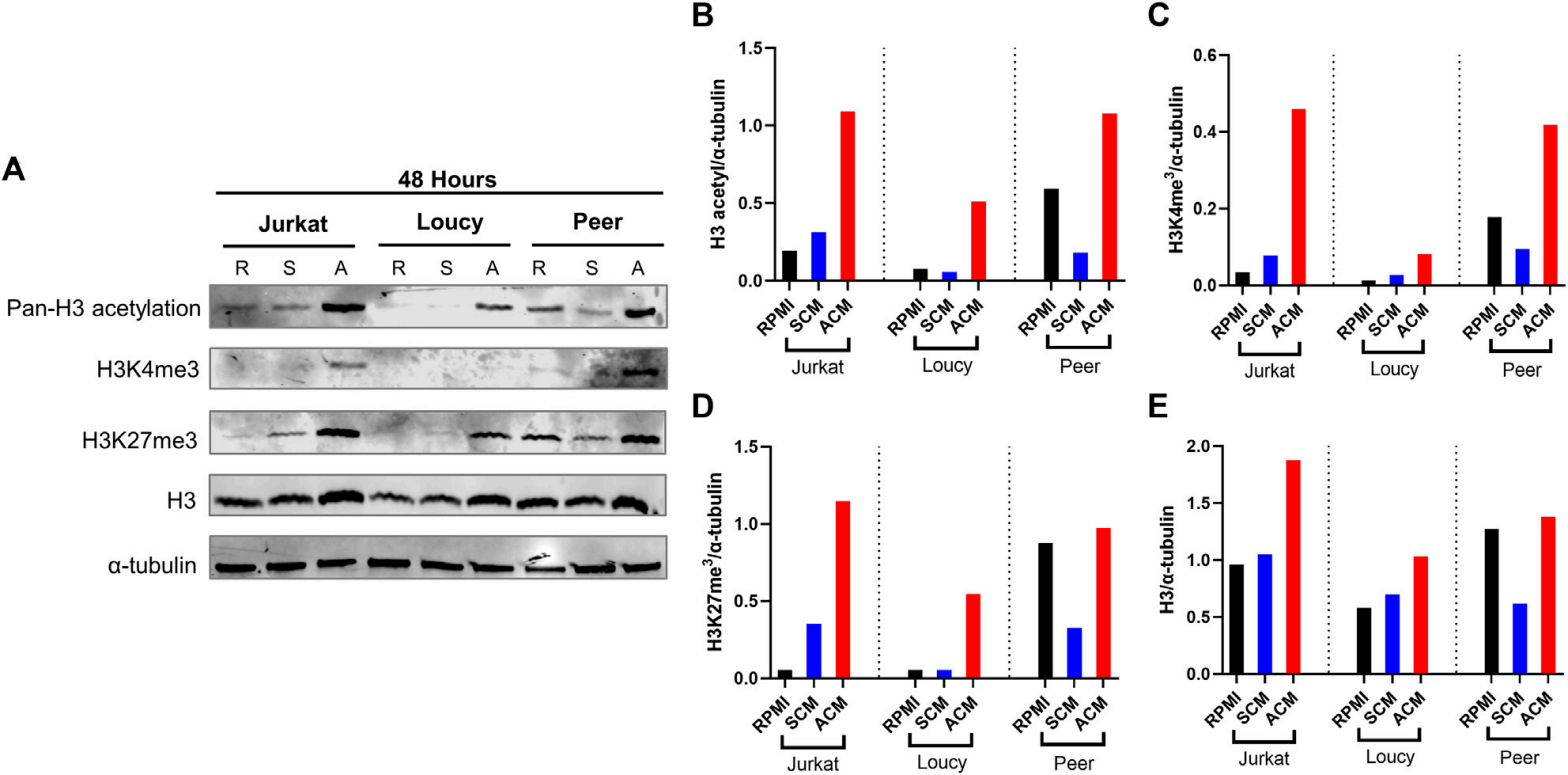
Adipocyte secretome alters the epigenome of human T-ALL cells. Human T-ALL cells (Jurkat, Loucy, and Peer) were cultured in unconditioned media, SCM, or ACM for 48 h, and Western blot analysis was conducted to determine the levels of modifications on histone 3 (H3) and total H3 levels. **(A)** Primary data from a Western blot experiment are shown. **(B–E)** Protein expression levels from Western blot experiments were determined using ImageJ analysis, and a representative blot from two independent experiments is shown.

Given the extensive gene expression changes associated with transcription, alterations in epigenetic modifiers, and histones in human T-ALL cells cultured in ACM relative to the other conditions tested (Supplementary Figure S3; Figure 2), we next determined how the adipocyte secretome impacted the nuclear chromatin landscape of human T-ALL cells. Adipocyte-mediated changes occurred very quickly in human T-ALL cells cultured in ACM relative to the other conditions tested. Culturing human T-ALL cells in ACM for 24 and 48 h led to a modest increase in the total H3 protein levels compared to the SCM and untreated conditions (Figures 4A–E; Supplementary Figure S7A). This result is consistent with replication-dependent histone biosynthesis (Armstrong and Spencer 2021). In addition, we observed 2- to 4-fold increases in transcriptional activation-associated histone posttranslational modifications (Hyun et al., 2017) on H3 (Figures 4A–E; Supplementary Figure S7A). These included increased acetylation (K9ac/K14ac/K18ac/K23ac/K27ac) and methylation (K4me3 and K27me3) PTMs on H3 in ACM-treated T-ALL cells compared to the control conditions. Histone H3 methylated at lysine 27 is associated with bivalent chromatin and transitional gene expression states (Kinkley et al., 2016) and gene silencing (Pan et al., 2018). Despite multiple cellular functions attributed to increased methylation on H3 at K4 and K27, our results demonstrate that adipocyte-secreted factors induce an epigenetic transition toward increased gene transcription (Supplementary Figure S3; Figure 2; Tables 3, 4).

To determine the mechanism of the adipocyte-mediated epigenetic fluctuations in human T-ALL cells, we investigated whether epigenetic machinery, chromatin-modifying enzymes, and structural proteins were altered in malignant T cells cultured in ACM. To this end, we tested the hypothesis that the increase in histone acetylation was accompanied by increased histone acetyltransferase (HAT) activity and/or lower histone deacetylation (HDAC). However, we observed no significant differences in HAT and HDAC activity in any of the cell lines, except increased HDAC activity in Jurkat T-ALL cells (Supplementary Figures S7C,D). To investigate the cause of increased H3K27me3, we measured changes in the histone methyltransferase complex polycomb repressive complex 2 (PRC2) *via* Western blot of EED, EZH2, and SUZ12 (Lehmann et al., 2012). We neither saw significant differences in PRC2 levels nor did we observe any changes in the chromatin proteins from the gene silencing complex PRC1 (BMI1 and RING1A) (Supplementary Figure S7B). These results demonstrate that increased H3K27me3 in adipocyte-exposed human T-ALL cells is not driven by changes in total PRC complex proteins; however, it is possible that adipocyte change the enzymatic activity of the PRC or its association with chromatin. Given the lack of detectable changes in the chromatin-modifying machinery, it is also possible that changes in the availability of epigenetic substrates (e.g., acetyl-CoA and SAM) might underlie ACM-induced epigenetic changes in human T-ALL cells (Phan et al., 2017). In all, these results demonstrate that the adipocyte secretome increases acetylation and methylation on H3, despite the induction of the inhibitory H3K27me3 PTM, augments transcription in malignant T cells.

### Epigenetic modifying drugs phenocopy cell cycle and epigenetic changes induced by adipocyte-conditioned media in human T-ALL cells

**FIGURE 5:**
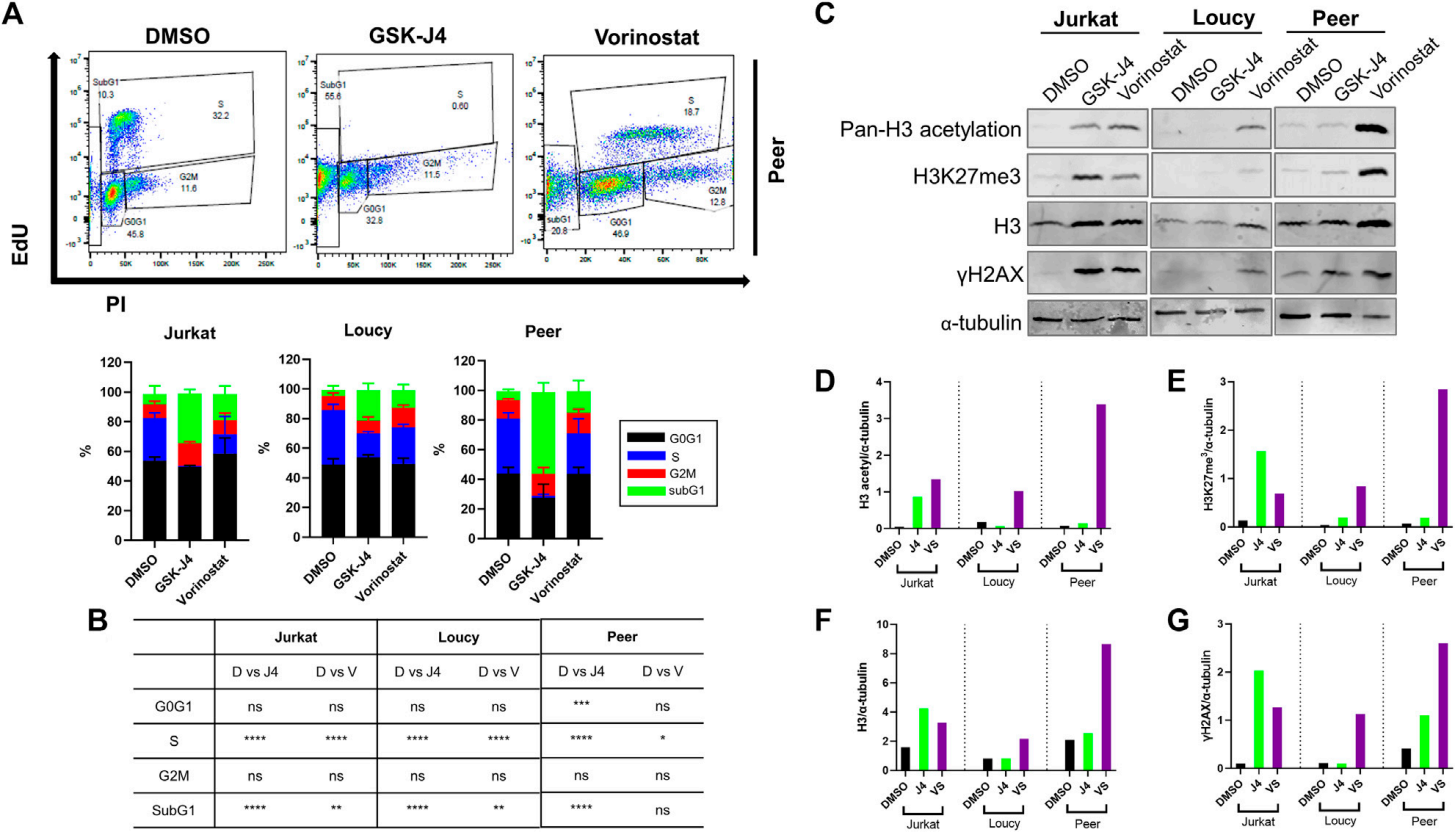
Epigenetic modifying drugs phenocopy cell cycle and epigenetic changes induced by ACM in human T-ALL cells. Human T-ALL cells (Jurkat, Loucy, and Peer) were treated with DMSO (control), GSK-J4 (a histone demethylase inhibitor), or vorinostat (a histone deacetylase inhibitor) for 72 h, and cell cycles were assessed using EdU analysis and flow cytometry. **(A)** Representative primary data from three independent experiments (top panel) and **(B)** cell cycle distribution data (bottom panel) from the combined experiments are shown. **(C)** Cells treated in **(A)** were processed for Western blot analysis to determine the levels of modifications on histone 3 (H3), total H3 levels, and indicators of genotoxic stress (γH2AX). **(D–G)** Protein expression levels from the Western blot experiments were determined using ImageJ analysis and a representative blot from two independent experiments is shown.

To determine whether the relationship between changes in histone modifications and genomic stability in human T-ALL was causal, we sought to determine if treating human T-ALL cells with epigenetic modifying drugs phenocopied ACM-induced genotoxic stress in malignant T cells. For these experiments, we tested how epigenetic modifying drugs, which increase acetylation and methylation (Supplementary Figure S8A) altered T-ALL cell cycle progression, PTMs on H3, and the induction of DNA damage. Upon determining the IC_50_ of GSK-343 (a histone methyltransferase inhibitor), GSK-J4 (a histone demethylase inhibitor), C646 (a histone acetyltransferase inhibitor), and vorinostat (a histone deacetylase or pan-HDAC inhibitor), we decided to move forward with testing the effects of GSK-J4 and vorinostat due to their low IC_50_ values (Supplementary Figures S8B–F). Similar to the impact of ACM on human T-ALL cells, GSK-J4 and vorinostat augmented cell cycle progression leading to a significant increase in cells in the SubG1 phase of the cell cycle (Supplementary Figure S9A; Figures 5A,B), which is indicative of cell death (Supplementary Figures S8B–F, S9B).

Given that treating human T-ALL cells with GSK-J4 and vorinostat promoted cell cycle progression similar to responses observed when malignant T cells were cultured in ACM, we next assessed the impact of these treatments on H3 acetylation and methylation by determining their on-target effects. Both drugs performed as expected when human T-ALL cells were treated; GSK-J4 increased methylation and vorinostat augmented acetylation in human malignant T cells (Supplementary Figure S9C; Figures 5C–E). Interestingly, similar to responses observed in ACM-treated T-ALL cells, both drugs also increased H3 protein levels and promoted DNA damage (γH2AX) in human T-ALL cells (Supplementary Figure S9C; Figures 5C,F,G). Taken together, these results reveal that genotoxic stress induced in human T-ALL cells after treatment with GSK-J4 and vorinostat results from increased acetylation or methylation on H3, which mimics epigenetic alterations observed in ACM-treated T-ALL cells.

### The adipocyte secretome augments the cytotoxic effects of epigenetic modifying drugs

Given that the adipocyte secretome and epigenetic modifying drugs promoted similar epigenetic changes and genotoxic stress in human T-ALL cells, we hypothesize that the adipocyte secretome would sensitize malignant T cells to the cytotoxic effects of epigenetic drugs.

**FIGURE 6:**
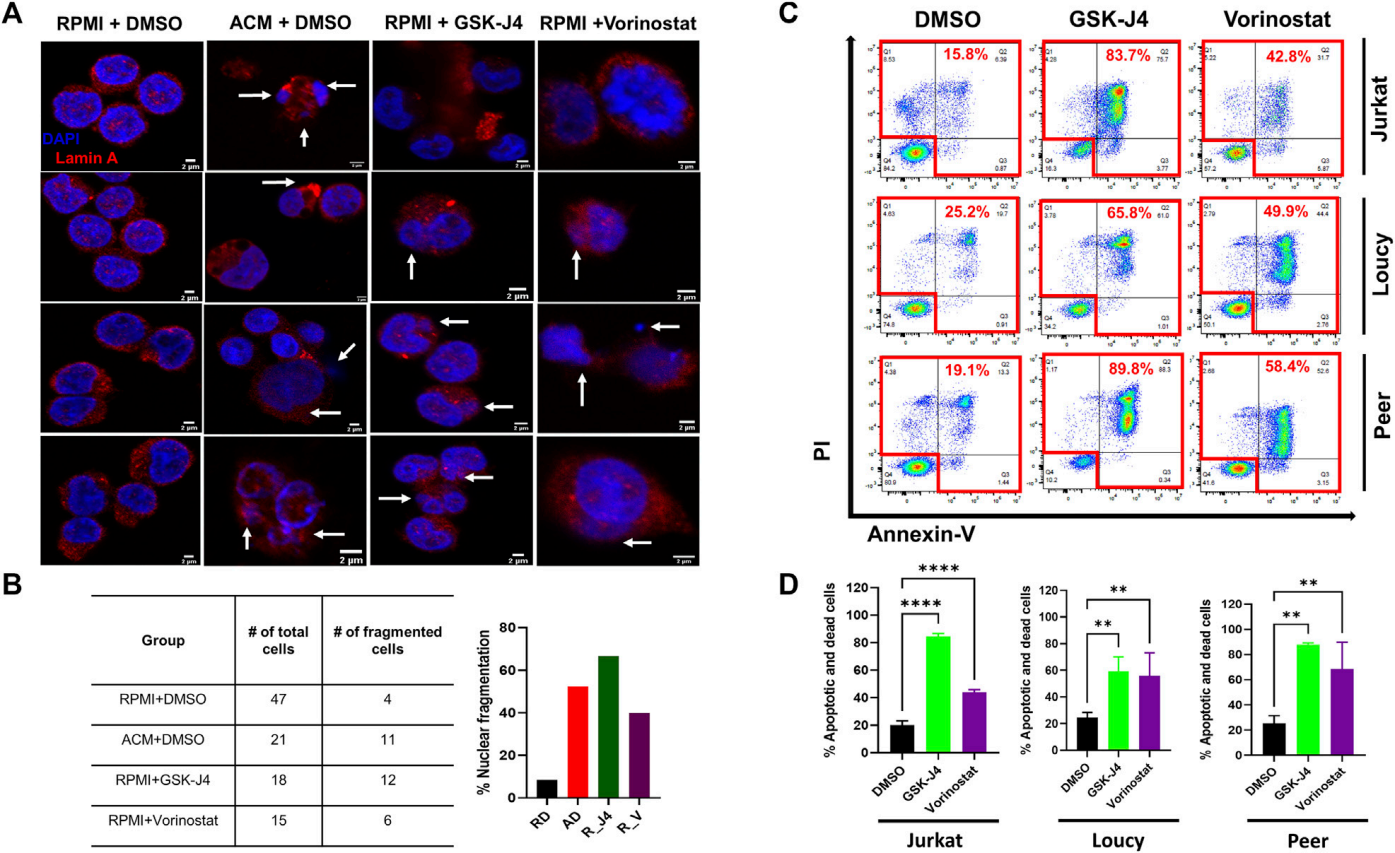
Treating human T-ALL cells with GSK-J4 and vorinostat phenocopy ACM-induced DNA damage and cytotoxicity in leukemia cells. Jurkat T cells were cultured in RPMI + DMSO, ACM + DMSO, or with RPMI + epigenetic modifying drugs (GSK-J4 or Vorinostat) for 48 h. The cells were then stained with lamin A with DAPI to visualize nuclei. **(A)** Representative images are shown with white arrows indicating nuclei spillage or fragmented nuclei. The percentage of cells harboring fragment nuclei, calculated by dividing the # of cells containing fragmented nuclei/total # of cell counted, is shown in **(B)**. **(C,D)** Human T-ALL cells (Jurkat, Loucy, and Peer) were treated with DMSO (control), GSK-J4 (a histone demethylase inhibitor), or vorinostat (a histone deacetylase inhibitor) for 72 h. The percentage of dead cells after 3 days of treatment was determined using Annexin-V/PI staining flow by flow cytometry. Representative primary data from one of three independent experiments are shown in **(C)** with quantitative data from combined experiments presented in **(D)**. Statistical significance was calculated using a one-way ANOVA followed by Tukey’s multiple comparison post-test. **p < 0.01 and ****p < 0.0001.

To further assess genomic alterations induced by the adipocyte secretome and epigenetic modifying drugs, we performed a nucleosome protection assay, in which DNA was isolated from human T-ALL cells cultured in unconditioned media, SCM, ACM, or cells cultured with the histone demethylase inhibitor GSK-J4 or HDAC inhibitor vorinostat, treated with nuclease, and run on an agarose gel to elevate chromatin fragmentation in malignant T cells. These experiments revealed that ACM induced the most significant amount of DNA fragmentation relative to cells cultured in unconditioned media or SCM (Supplementary Figure S10A). Albeit to a lesser extent, treating human T-ALL cells with GSK-J4 and vorinostat resulted in a similar response, further demonstrating that both an adipose-rich microenvironment and epigenetic modifying drugs induce genomic alterations in human T-ALL cells (Supplementary Figure S10A). In further support of this conclusion, confocal analyses of DNA integrity in human T-ALL cells under each condition revealed that ACM, GSK-J4, and vorinostat induced similar amounts of nuclear fragmentation (abnormal nuclei as determined by dysmorphic DAPI staining, nuclei shrinkage/condensation, or spillage of the nuclear protein Lamin A) in malignant T cells (Figures 6A,B).

Given our results demonstrating that epigenetic instability and genotoxic stress induce cell death in human T-ALL cells treated with ACM, we next determined the extent of cytotoxicity induced by single-agent treatments with GSK-J4 and vorinostat in malignant T cells. After 3 days of culture, we found that GSK-J4 treatment induced between 45 and 85% cell death in human T-ALL cells (Supplementary Figures S8B–D, S9B; Figures 6C,D) and treatment with vorinostat induced between 20 and 90% cell death in human T-ALL cells (Supplementary Figure S8B,E,F, S9B; Figures 6C,D). The amount of T-ALL cell death over 3 days of culture was largely dependent on the differentiation state of the leukemia cell. Notably, the cytotoxic effects of ACM, and single-agent GSK-J4, vorinostat, and C646 treatment was specific to human T-ALL cells, given that we did not observe cell death in non-malignant murine (Supplementary Figure S11) or human (Supplementary Figure S12) T cells when exposed under either condition.

**FIGURE 7:**
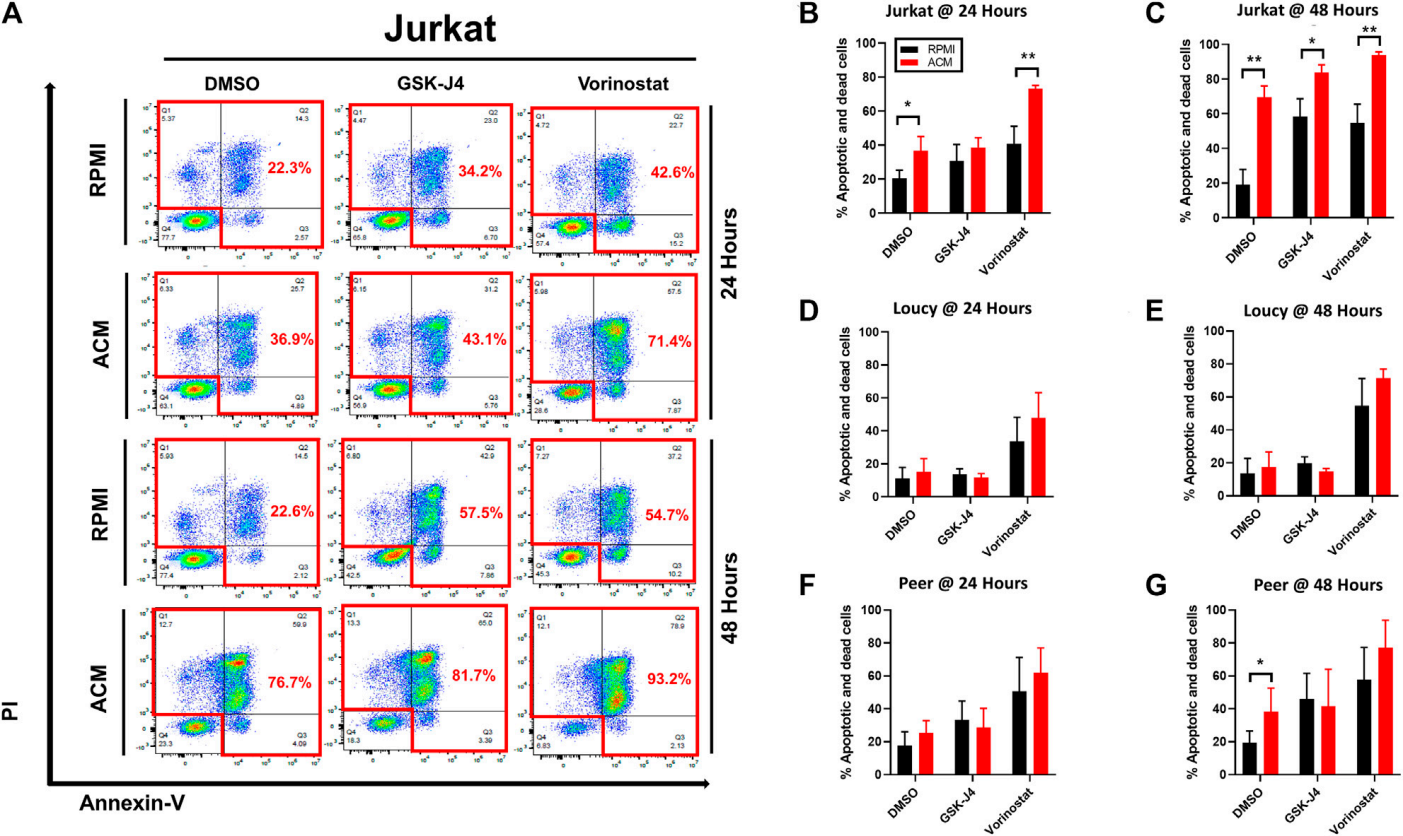
Adipocyte secretome augments the cytotoxic effects of epigenetic modifying drugs. Human T-ALL cells (Jurkat, Loucy, and Peer) were cultured in either RPMI or ACM with or without the epigenetic modifying drugs GSK-J4 or vorinostat for 24 and 48 h. T-ALL cell death was determined using Annexin-V/PI staining followed by flow cytometric analysis at both time points. **(A)** Annexin-V/PI flow cytometry data are shown for a representative of three independent experiments. **(B–G)** Quantitative data from combined experiments (*n* ≥ 3) are presented. A Student’s *t*-test was performed to determine statistical significance in **(B–G)**. *p < 0.05 and **p < 0.01.

We next determined if the adipocyte secretome sensitized human T-ALL cells to the cytotoxic effects of epigenetic modifying drugs. In most cases we observed that leukemia cells cultured in ACM were sensitized to the cytotoxic effects of epigenetic modifying drugs within 24–48 h (Figures 7A–G). Notably, this effect was more pronounced in the more differentiated Jurkat and Peer T-ALL cell lines, where cytotoxicity ranged from 40 to 95% over 2 days of culture for GSK-J4 and vorinostat (Figures 7A–G). Furthermore, the ACM-mediated effect was more pronounced in Jurkat T cells after 3 days of culture as cytotoxicity exceeded 90% in all cases (Supplementary Figure S10B). We are currently in the process of identifying the adipocyte-secreted factors, which induce human T-ALL cell death. We have performed mass spectrometry analysis of the adipocyte secretome and have identified candidates such as vascular non-inflammatory molecule 3 (VNN3), an ectoenzyme which induces inflammation (Wang N et al., 2018), as a potential contributor to apoptosis (data not shown).

## Discussion

Obesity rates are increasing globally, with the United States being particularly affected by this pandemic (Finkelstein et al., 2012). In the United States, people with obesity are increasing in both adult (>19 years old) and pediatric (ages 2–19 years old) populations (Ogden et al., 2014; Andolfi and Fisichella 2018). In spite of the fact that obesity is a risk factor for developing and succumbing to many cancers (Butturini et al., 2007), these associations are still controversial for some cancer types such as T-ALL (Heiblig et al., 2015).

Using murine models of obesity, we present data in support of previously published epidemiological studies demonstrating that obesity is protective against T-ALL pathogenesis in pediatric populations (Heiblig et al., 2015). In both syngeneic and xenograft studies, obese mice exhibited a 20%–60% greater chance of surviving over 3 months post-leukemia cell transplantation relative to their lean counterparts. Furthermore, we demonstrate that the adipocyte secretome is highly cytotoxic to human T-ALL cells, which was not dependent on the p53 mutation status. Interestingly, the kinetics of apoptosis varied between more and less differentiated T-ALL cells, with extensive cell death being observed rapidly (2–3 days) for the more differentiated T-ALL cells, while the phenotypically immature T-ALL cells reached similar levels of cytotoxicity after 5 days of culture.

Mechanistically, we found that the adipocyte secretome promoted extensive gene expression changes in human T-ALL cells, which was highlighted by the activation of cell cycle, gene transcription, DNA replication, and DNA repair pathways. These alterations were associated with increased cell cycle progression with accompanying DNA damage, which could be partially explained by decreases in gene and protein levels of cell cycle regulators (notably *CHEK1* and *CHEK2*), although the activation of these mediators was elevated in human T-ALL cells cultured in adipocyte-secreted factors. In addition to reducing *CHEK1* and *CHEK2* gene expression levels, the adipocyte secretome also suppressed *BMI1* transcription in more differentiated T-ALL cells as demonstrated by rapid death of human T-ALL cells when cultured in the adipocyte secretome. This is notable due to BMI1-mediated suppression of the *INK4A*/*ARF* locus and warrants further investigation into the regulation of this tumor suppressor in T-ALL cells exposed to adipocyte-secreted factors.

In addition, the phenotypic and functional changes in human T-ALL cells were accompanied by increases in H3 protein levels and selective posttranslational modifications of H3 (acetylation and tri-methylation of K4 and K27), which were indicative of gene activation and repression occurring in ACM-cultured human T-ALL cells. From our RNA-sequencing studies, we concluded that the modifications resulting in activation dominated in human T-ALL cells cultured in ACM, given our pathway analysis data demonstrating enhanced gene transcription and cell cycle progression in malignant T cells cultured in the adipocyte secretome.

Despite the epigenetic flux observed in ACM-stimulated human T-ALL cells, we did not observe increases in the detection of proteins, which compromise PRC1 (BMI1 and RING1A) or PRC2 (EZH2, EED, and SUZ12), which can monomethylate, dimethylate, and trimethylate H3K7 (Dobrinic et al., 2021). Histone 3 lysine 4 methylation is regulated by several histone methyltransferases [KMT2A-F; (Shilatifard 2012)] and demethylases [six known enzymes; (Hyun et al., 2017)]. Histone acetylation is also a highly dynamic process, which is regulated by 19 histone acetyltransferases (Roth et al., 2001) and 18 histone deacetylases (Bolden et al., 2006). To narrow down candidate epigenetic modifiers, which may be differentially regulated in ACM-exposed human T-ALL cells, we determined histone deacetylase (HDAC) and histone acetyltransferase (HAT) activities in malignant T cells cultured under each condition. Only one of the three human T-ALL cell lines exhibited a significant increase in HAT activity when cultured in ACM relative to the other conditions tested, while HDAC activity was elevated, albeit insignificantly, in the more differentiated human T-ALL cell lines. These results demonstrate that adipocyte-mediated PTM alterations on H3 in human T-ALL cells are neither driven by changes in total PRC complex proteins nor HDAC or HAT activity. Therefore, it is possible that the adipocyte secretome alters PRC assembly, its association with chromatin, or the availability of epigenetic substrates (e.g., acetyl-CoA and SAM) to mediated PTMs on H3 in human T-ALL cells and these possibilities are currently under investigation.

Given our findings that adipocyte-induced alterations in the T-ALL epigenome were associated with increased DNA damage and cell death, we also determined if inhibitors of enzymes regulating PTMs on H3 promote cell death in a similar manner. Indeed, we observed that epigenetic modifying drugs worked as expected with GSK-J4 and vorinostat increasing methylation and acetylation PTMs on H3 in drug-treated human T-ALL cells similar to profiles observed in ACM-cultured leukemia cells. Interestingly, we observed that GSK-J4 is cytotoxic to human T-ALL cells at dosages much lower (0.5–3 µM) than previously reported (2–10 µM) (Ntziachristos et al., 2014; Benyoucef et al., 2016), and we achieved ≥80% *in vitro* cytotoxicity if experiments were carried out for greater than 3 days (data not shown). Our results highlight the potency of GSK-J4 against this leukemia subtype. Furthermore, inhibition of either HDACs or histone demethylases promoted DNA damage and were highly cytotoxic to human T-ALL cells. Given that both the adipocyte secretome and inhibition of chromatin-modifying enzymes promoted cytotoxicity in human T-ALL cells by similar epigenetic mechanisms, we hypothesized that we would observe enhanced killing of adipocyte-exposed human T-ALL cell treated with GSK-J4 or vorinostat. For these studies, we found that the more differentiated human T-ALL cells were killed faster by treatment with HDAC or histone demethylase inhibitors if cultured in the adipocyte secretome, which suggest that adipocyte-induced changes to the epigenome of human T-ALL cells can be exploited to enhance the potency of epigenetic modifying drugs.

The most common mutation in human T-ALL cells is a deletion of *CDKN2A,* which occurs in about 70% of cases (Hebert et al., 1994). A deletion in *CDKN2A* gene confers survival properties to malignant T cells due to the gene product promoting the expression of the tumor suppressors p16(INK4A) and p14(Arf), which maintain cells harboring DNA damage from growing and dividing very rapidly. Therefore, most human T-ALL cells present with elevated genotoxic stress due to deletions in CDKN2A. Gain-of-function mutations in *NOTCH1* are also commonly found in human T-ALL cells (Weng et al., 2004). These mutations promote increased CDK2 activity (Sarmento et al., 2005), and thus, cell cycle progression (Dohda et al., 2007). Due to increased proliferation and the metabolic demands associated with this phenotype, replicative and genotoxic stress is also a common feature of malignant T cells with this mutation. In addition, mutations in epigenetic modifiers are common in human T-ALL cells (Liu et al., 2017). Mutations in *EZH2,* the catalytic subunit of PRC2, are the most common epigenetic mutation found in T-ALL (Wang C et al., 2018). However, the impact of mutations in this complex is unknown in T-ALL, given that mutations in PRC2 are associated with transformation and it also has been identified as a tumor suppressor in certain cancers (Wang C et al., 2018).

Based on our data demonstrating that there is a delicate balance between increased proliferation and genotoxic stress in human T-ALL cells, and that these traits are commonly associated with mutations in epigenetic modifiers, it stands to reason that the latter property could be leveraged for therapeutic benefit. Indeed, vorinostat has shown preclinical efficacy in lean mice when combined with other treatment modalities for T-ALL (Gao et al., 2016; Jing et al., 2018) and was FDA-approved in 2006 for the treatment of cutaneous T cell lymphoma (CTCL) in patients with progressive, persistent, or recurrent disease (Chen et al., 2020). Despite the success of vorinostat in treating patients with CTCL, epigenetic modifying drugs are not FDA-approved to treat T-ALL. Encouragingly, preclinical assessment of the third-generation epigenetic modifying drugs ivosidenib (an isocitrate dehydrogenase 1 inhibitor) and tazemetostat (an EZH2 inhibitor) show enhanced potency and broader spectrum of cytotoxicity for hematological malignancies and solid cancers (Italiano et al., 2018).

Data presented in our study further support the continued exploration of inhibitors targeting chromatin-modifying enzymes as novel therapeutic strategies for T-ALL. In addition to vorinostat, we present data demonstrating that GSK-J4 and C646 induce significant cytotoxicity in human T-ALL cells at low micromolar concentrations. To our knowledge, data presented in our study represent the first reports of C646-mediated cytotoxicity of human T-ALL cells. This drug has also shown efficacy in models of AML, pancreatic, gastric, and cervical cancers at higher dosages (10–50 µM) than we observed in our study; however, preclinical dosages of 2 µM have been documented to translate to effective clinical responses (Gao et al., 2013; Ono et al., 2016; He et al., 2017; Wang et al., 2017; Ono et al., 2021). In addition to our results demonstrating that JMJD3/UTX and p300 inhibition may provide therapeutic benefit to patients with T-ALL, two additional manuscripts present preclinical evidence in support of this claim (Ntziachristos et al., 2014; Xia et al., 2019). Furthermore, to our knowledge, this study is the first to demonstrate that the small molecule inhibitor, C646, is capable of potently killing human T-ALL cells when used at clinically achievable doses. In contrast, GSK-343 required excessively high concentrations to kill human T-ALL cells; whereas, this small molecule inhibitor appears to be effective at killing solid cancers (Yu et al., 2017).

## Conclusion

**FIGURE 8:**
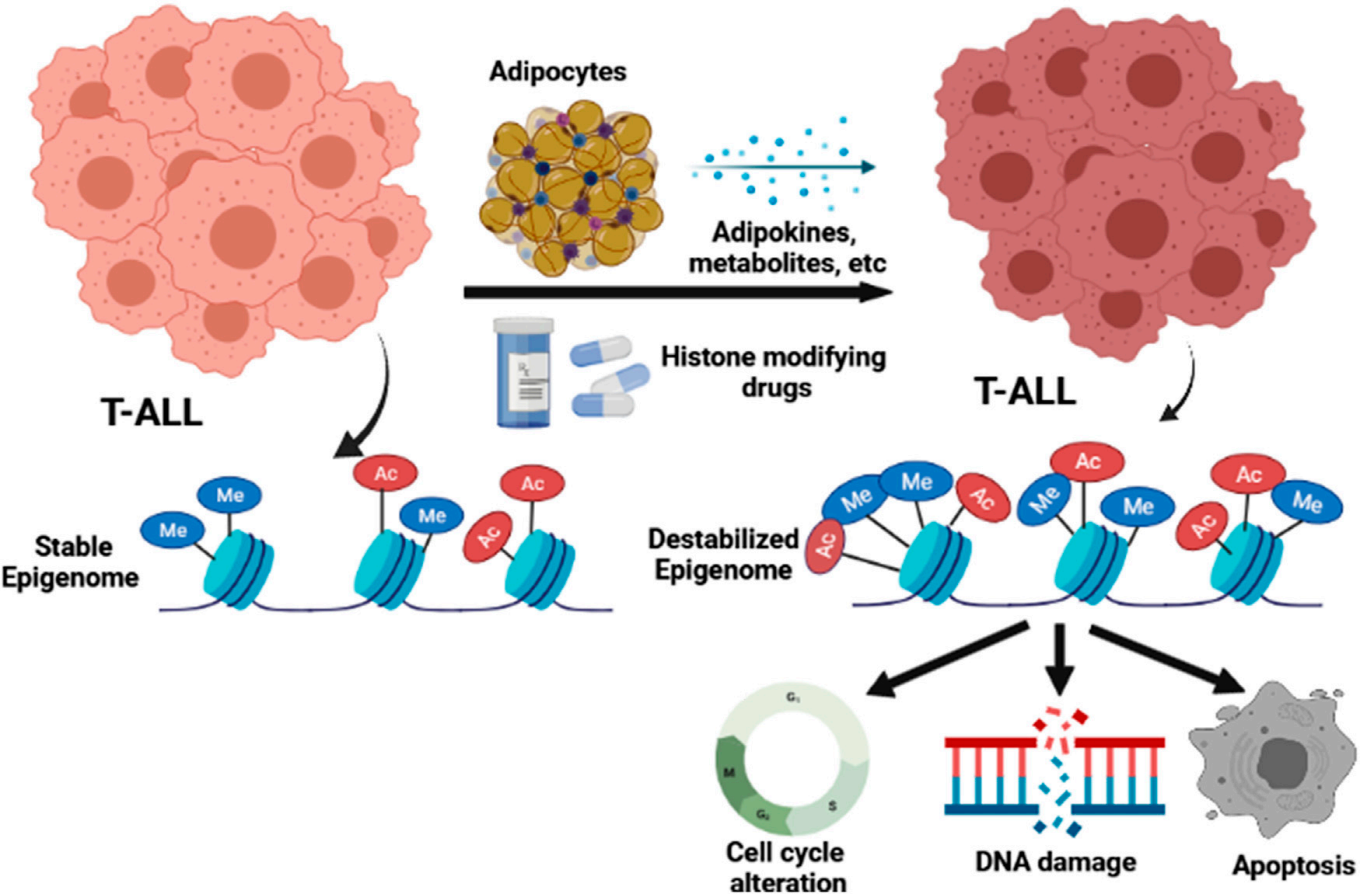
Model for how the adipocyte secretome and epigenetic modifying drugs impact the epigenome and function of human T-ALL cells.

In summary, our studies reveal that the inherent epigenetic instability in human T-ALL cells can be usurped to promote extensive leukemia cell death, and we present data demonstrating that this mechanism explains adipocyte-mediated protection against T-ALL pathogenesis. Furthermore, our data reveal that the adipocyte secretome and inhibitors that target the chromatin-modifying enzymes JMJD3/UTX and HDACs, induce similar epigenetic programs, which are highly cytotoxic to human T-ALL cells (Figure 8).

## Data Availability

The datasets generated during this study are available from the corresponding author upon request. The raw data and processed RNA-seq data used in this study are deposited in the GEO repository under accession number GSE202225 and can be accessed without restrictions (https://www.ncbi.nlm.nih.gov/geo/query/acc.cgi?acc=GSE202225).
