# Supplementary figures and images for: Adipocyte-mediated epigenomic instability in human T-ALL cells is cytotoxic and phenocopied by epigenetic-modifying drugs
Source: Front Cell Dev Biol. 2022 Aug 19;10:909557. doi: 10.3389/fcell.2022.909557 (PMC9438935; doi:10.3389/fcell.2022.909557)

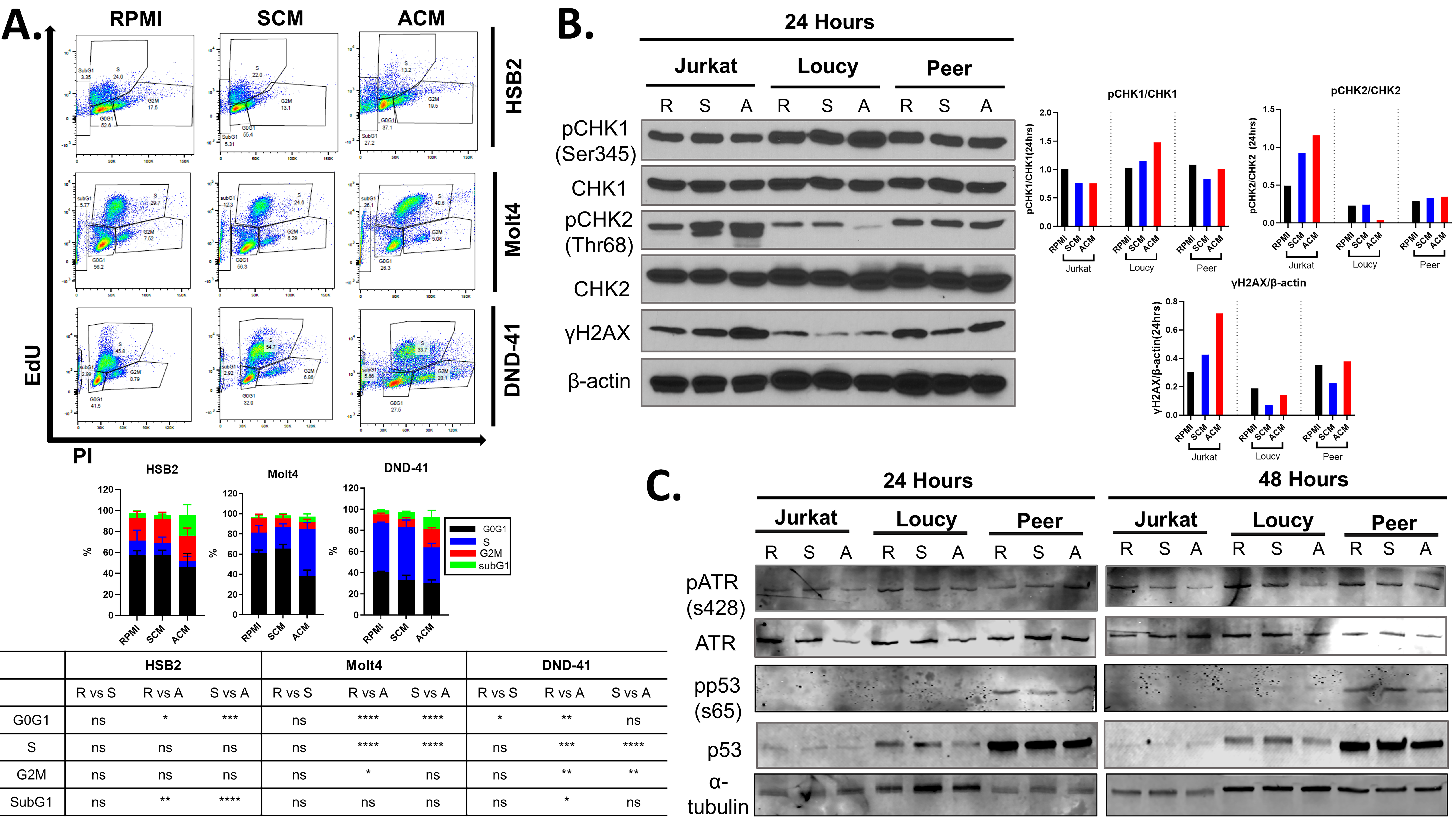

Supplement: Supplementary file 1 [file Image6.tif]

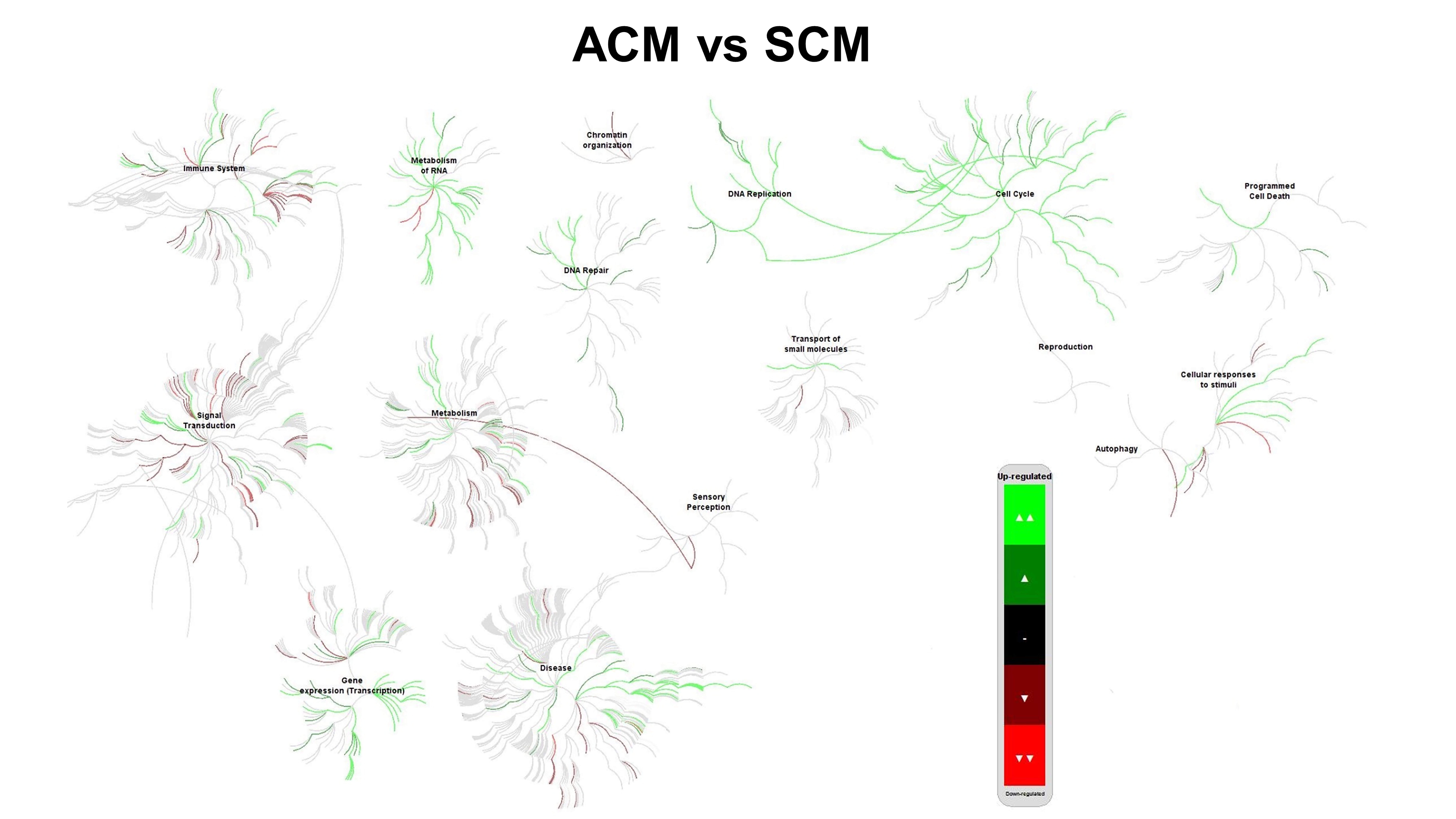

Supplement: Supplementary file 2 [file Image3.tif]

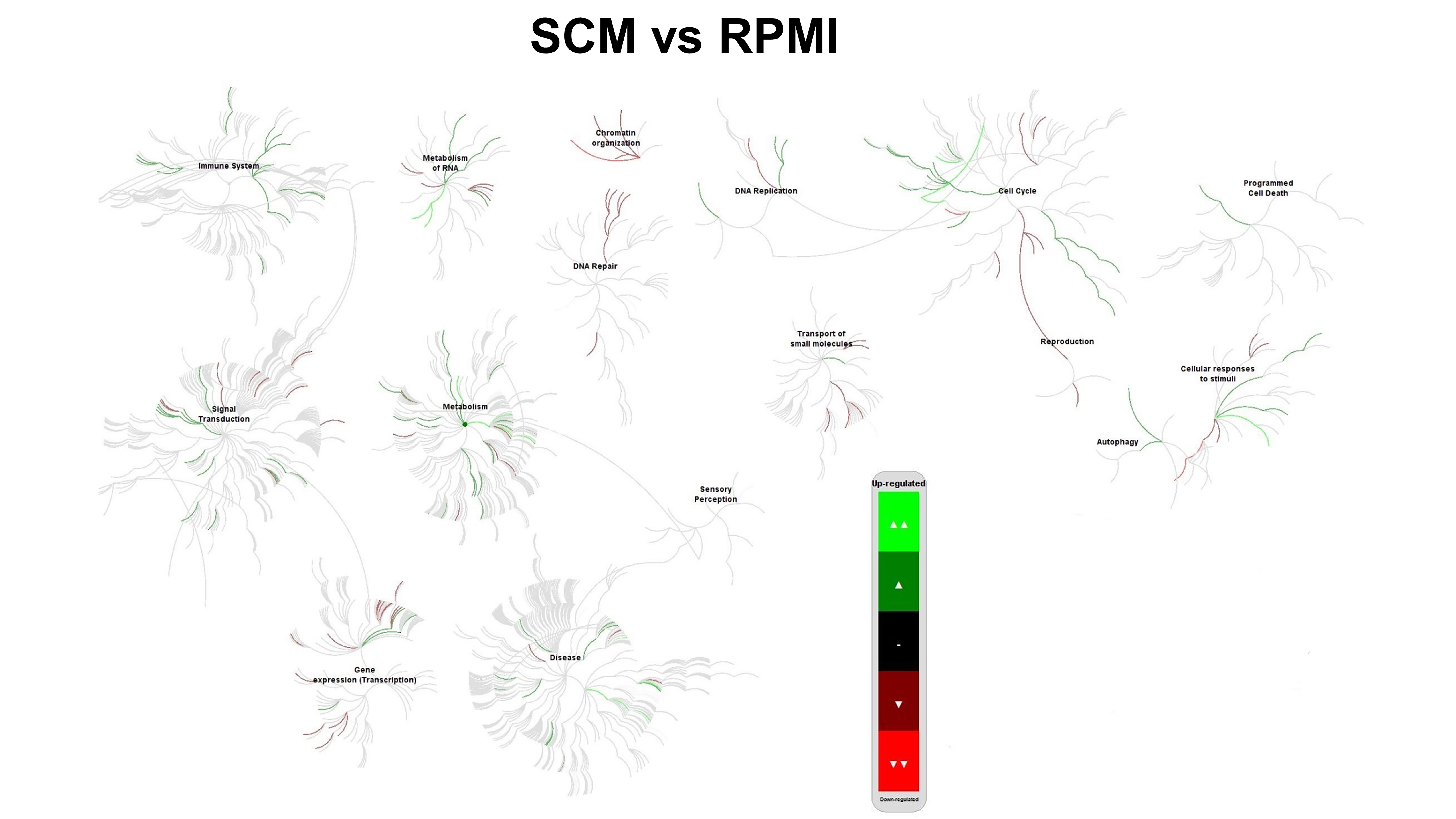

Supplement: Supplementary file 3 [file Image4.tif]

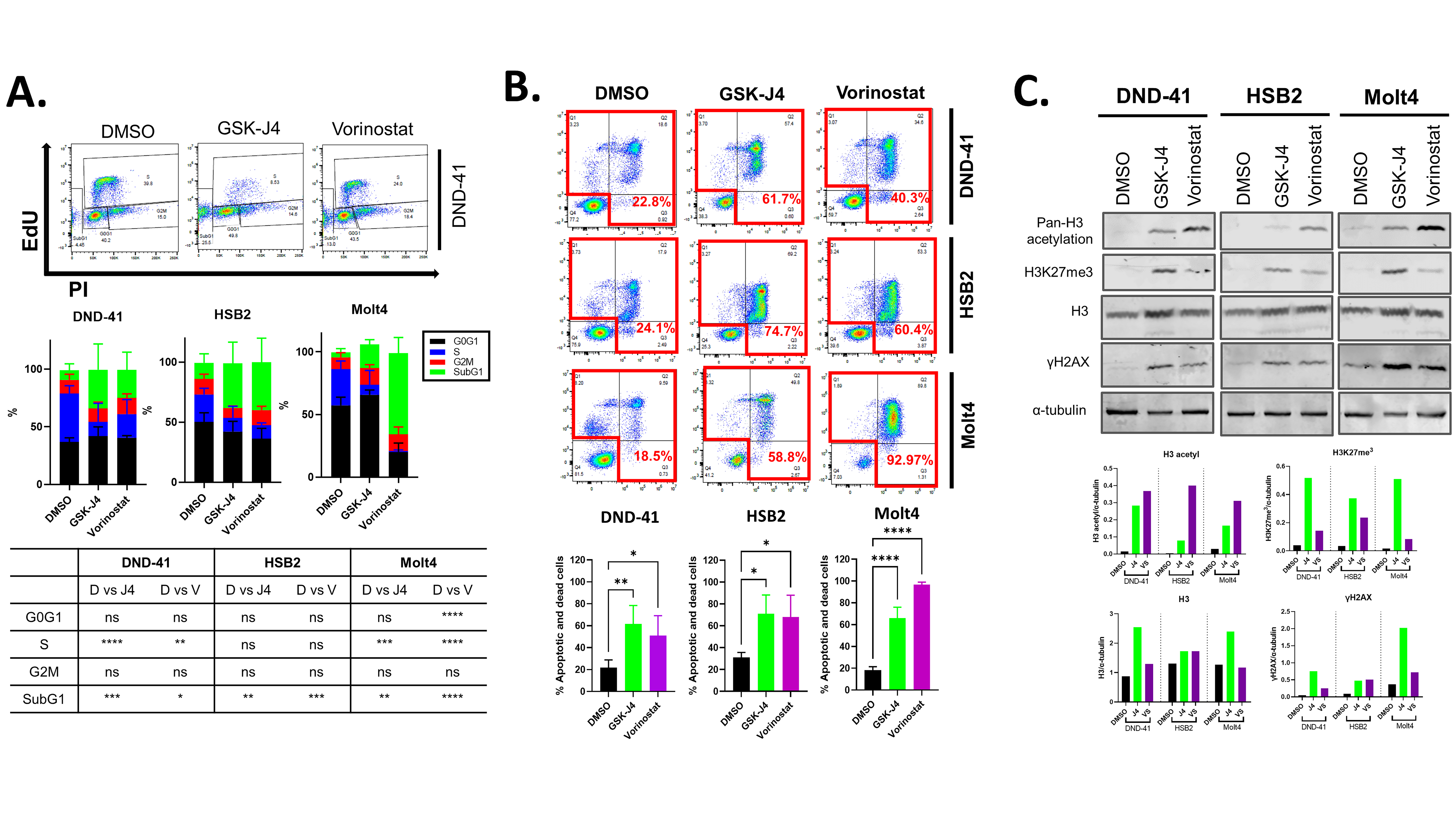

Supplement: Supplementary file 4 [file Image9.tif]

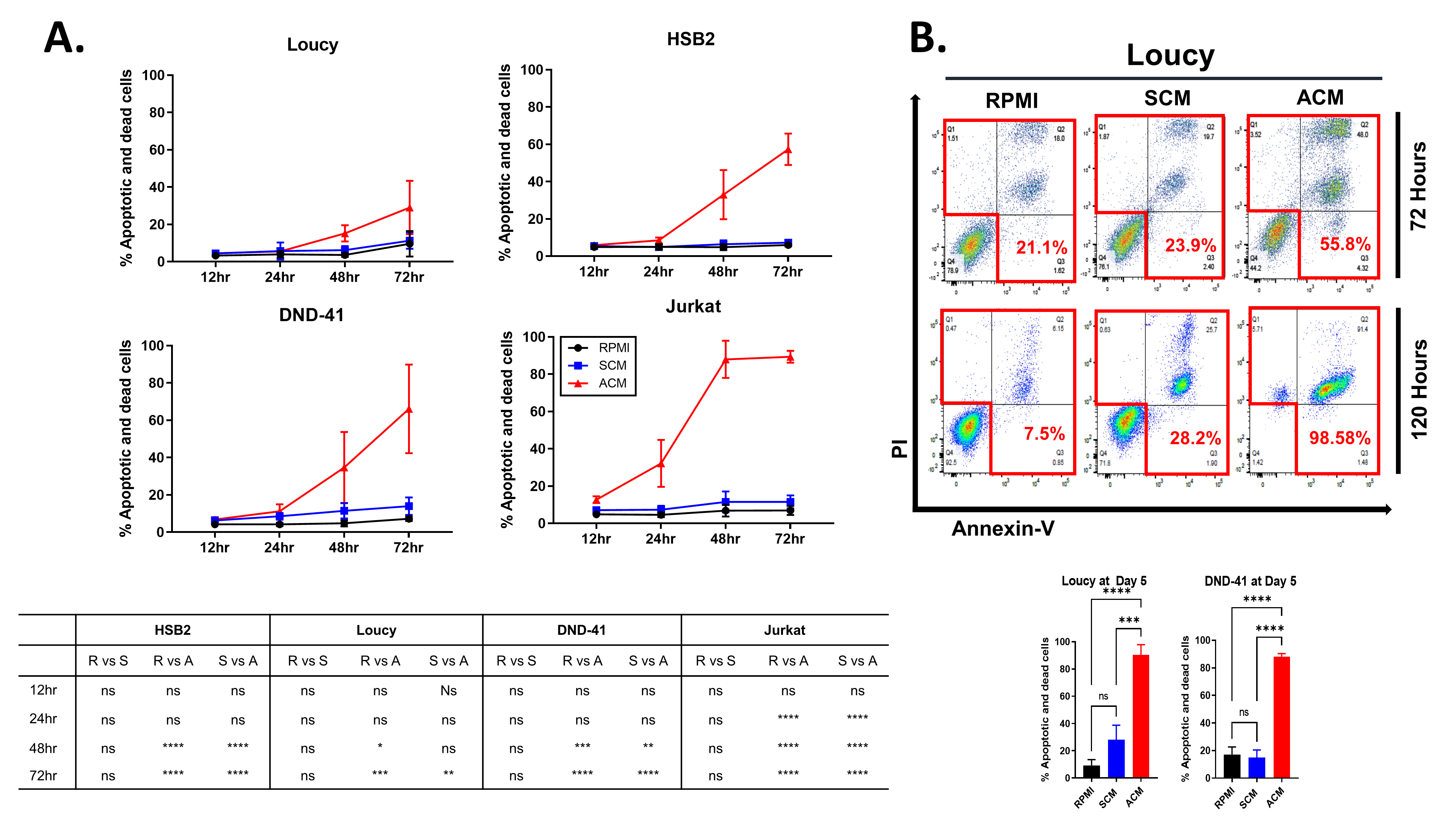

Supplement: Supplementary file 5 [file Image2.tif]

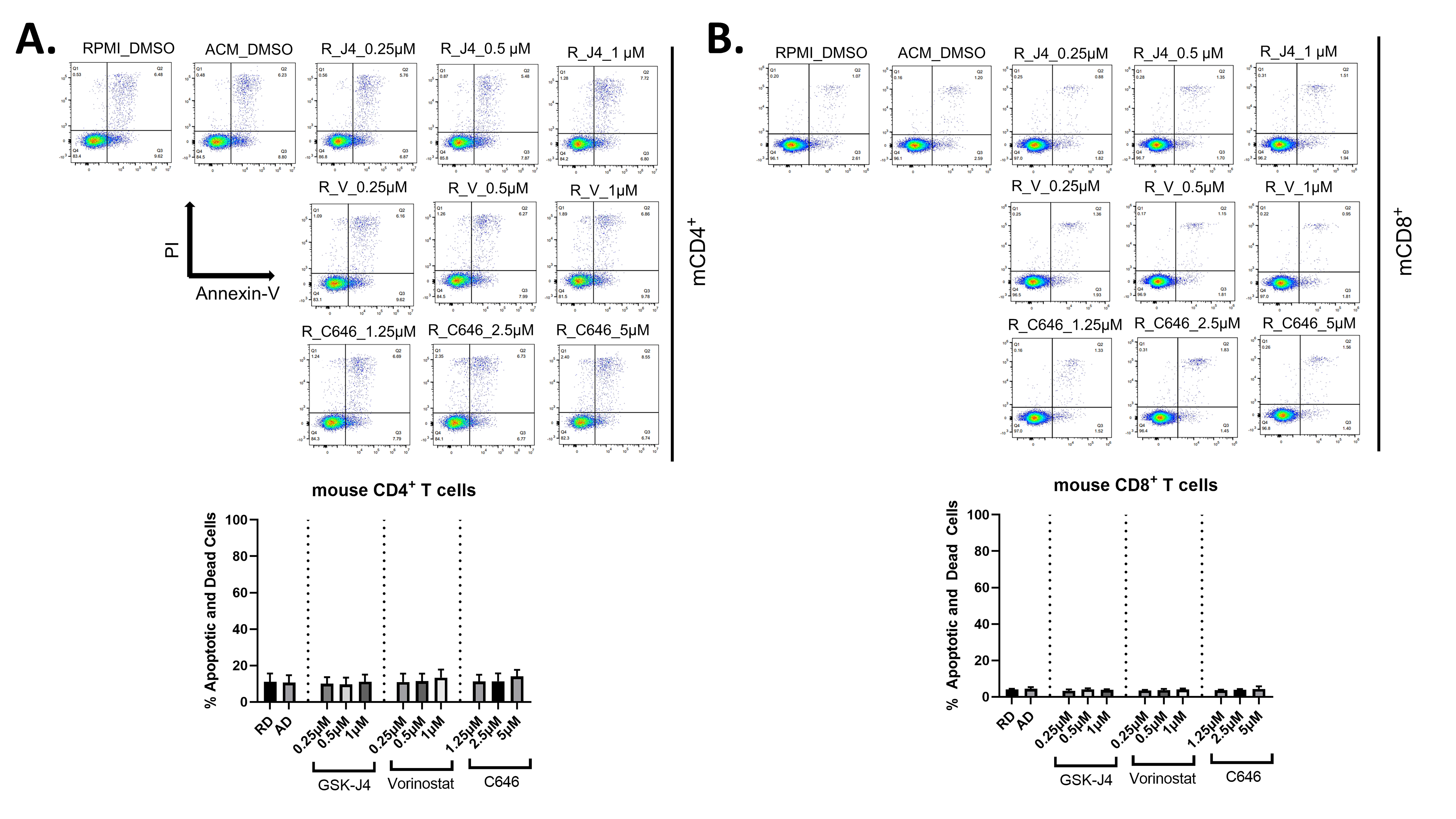

Supplement: Supplementary file 6 [file Image11.tif]

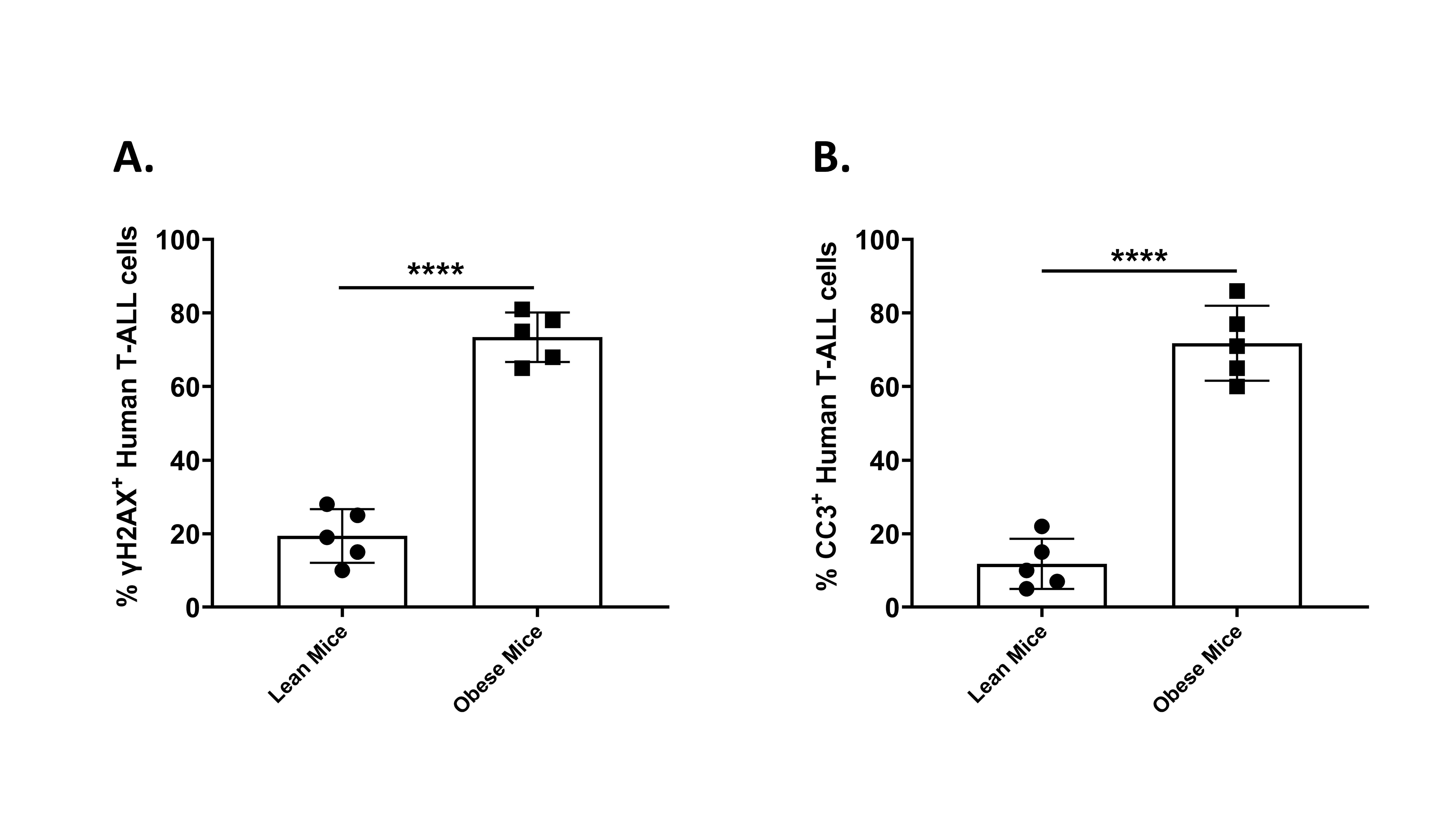

Supplement: Supplementary file 7 [file Image1.tif]

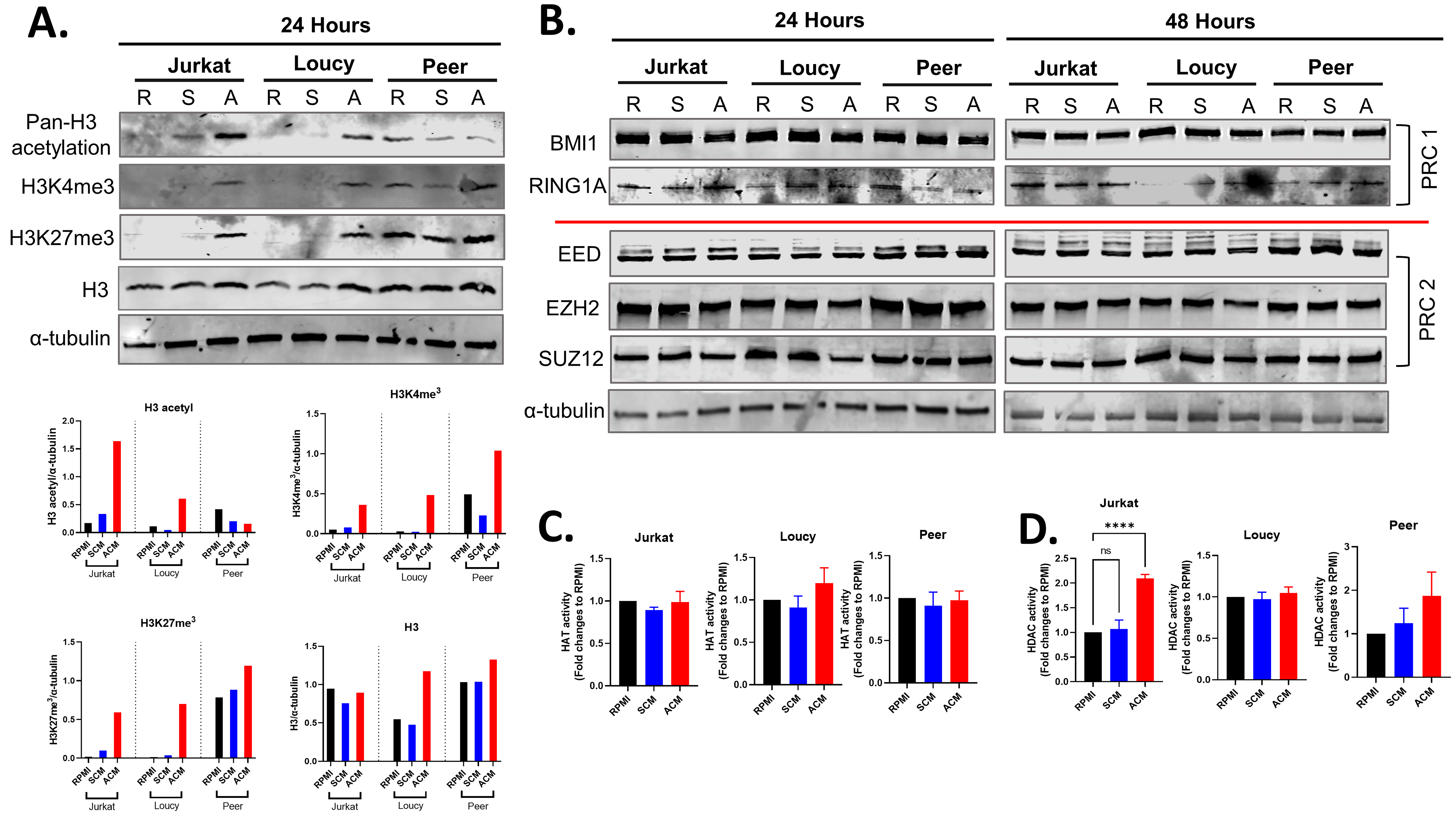

Supplement: Supplementary file 9 [file Image7.tif]

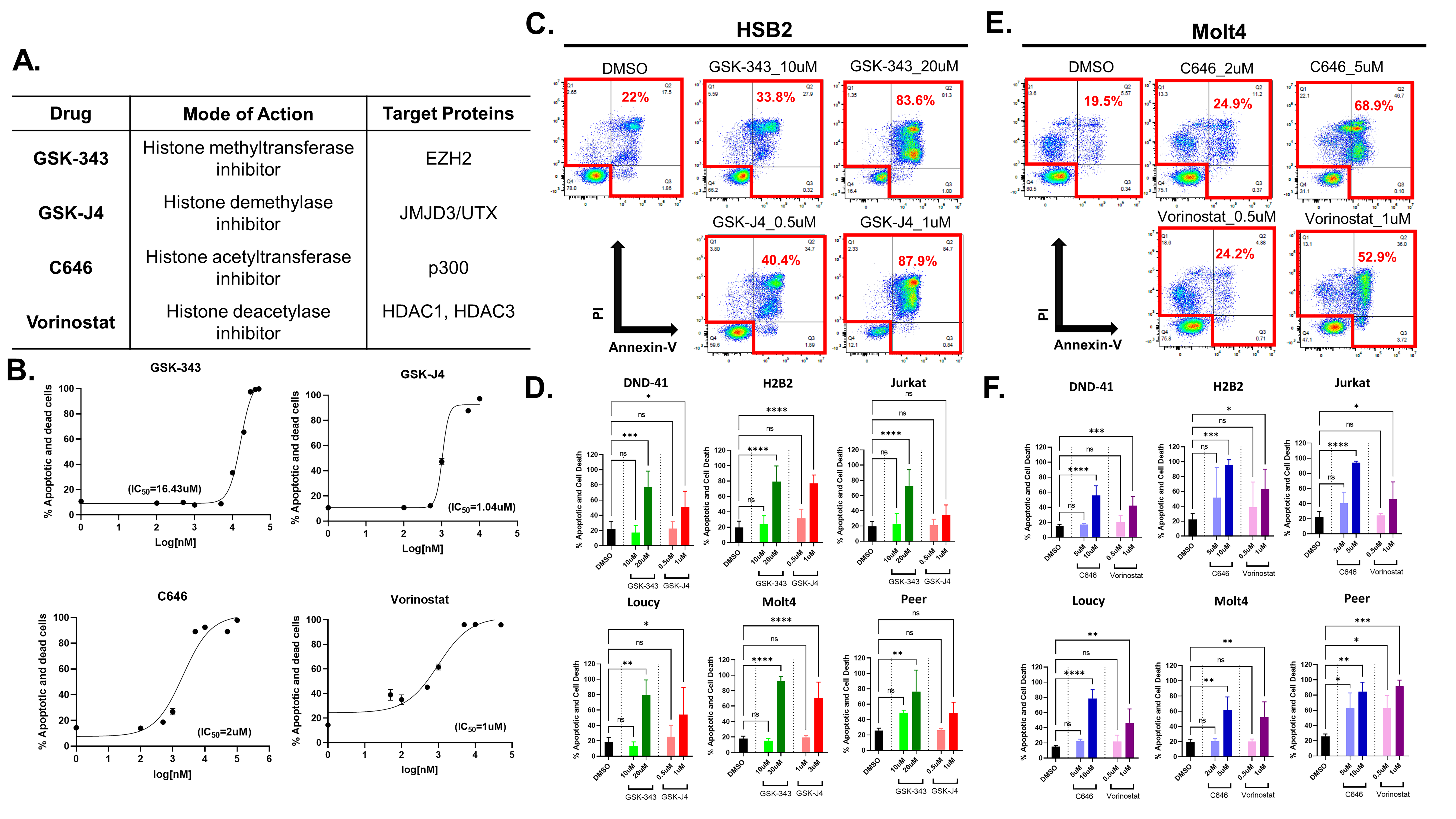

Supplement: Supplementary file 10 [file Image8.tif]

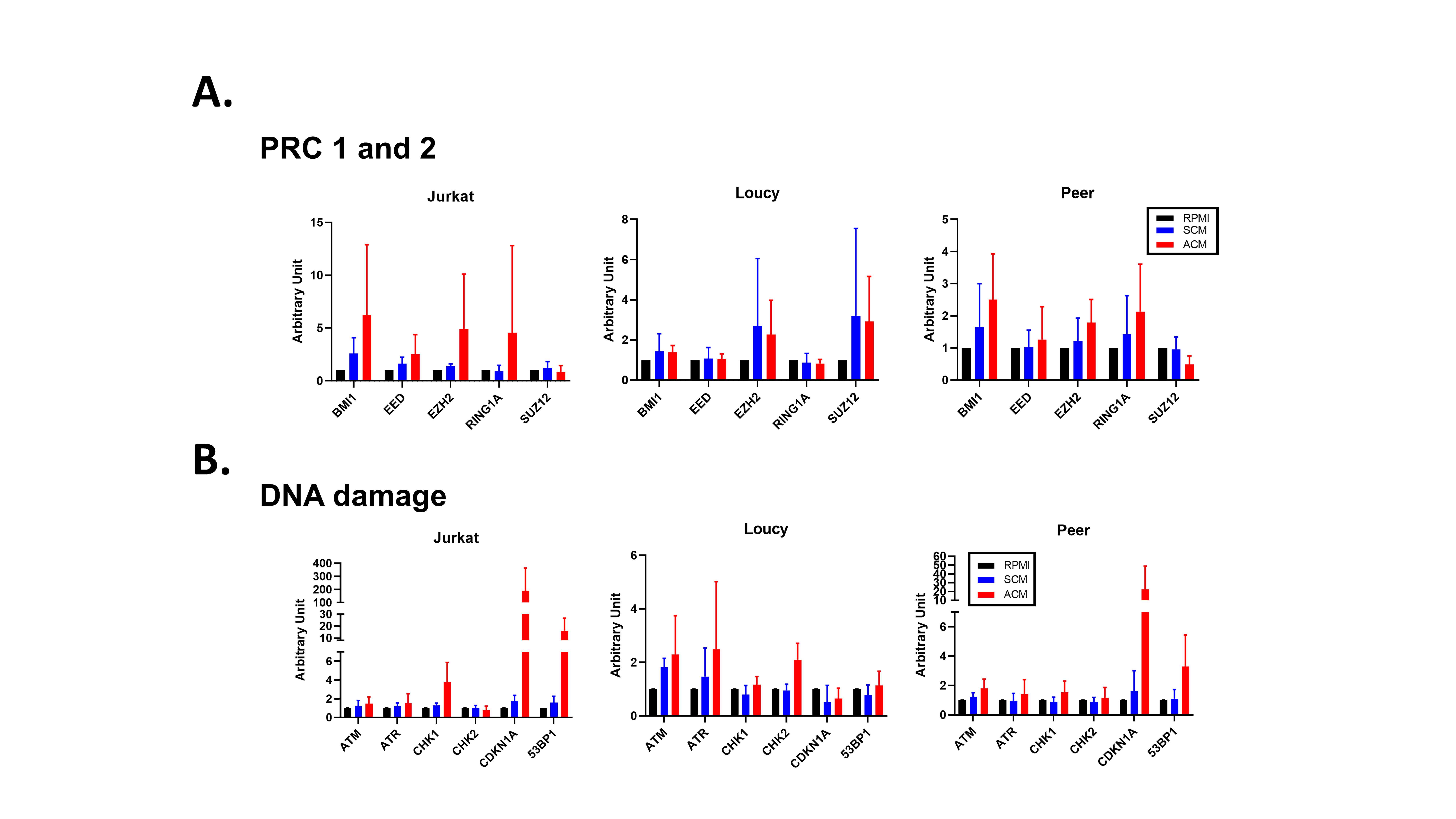

Supplement: Supplementary file 11 [file Image5.tif]

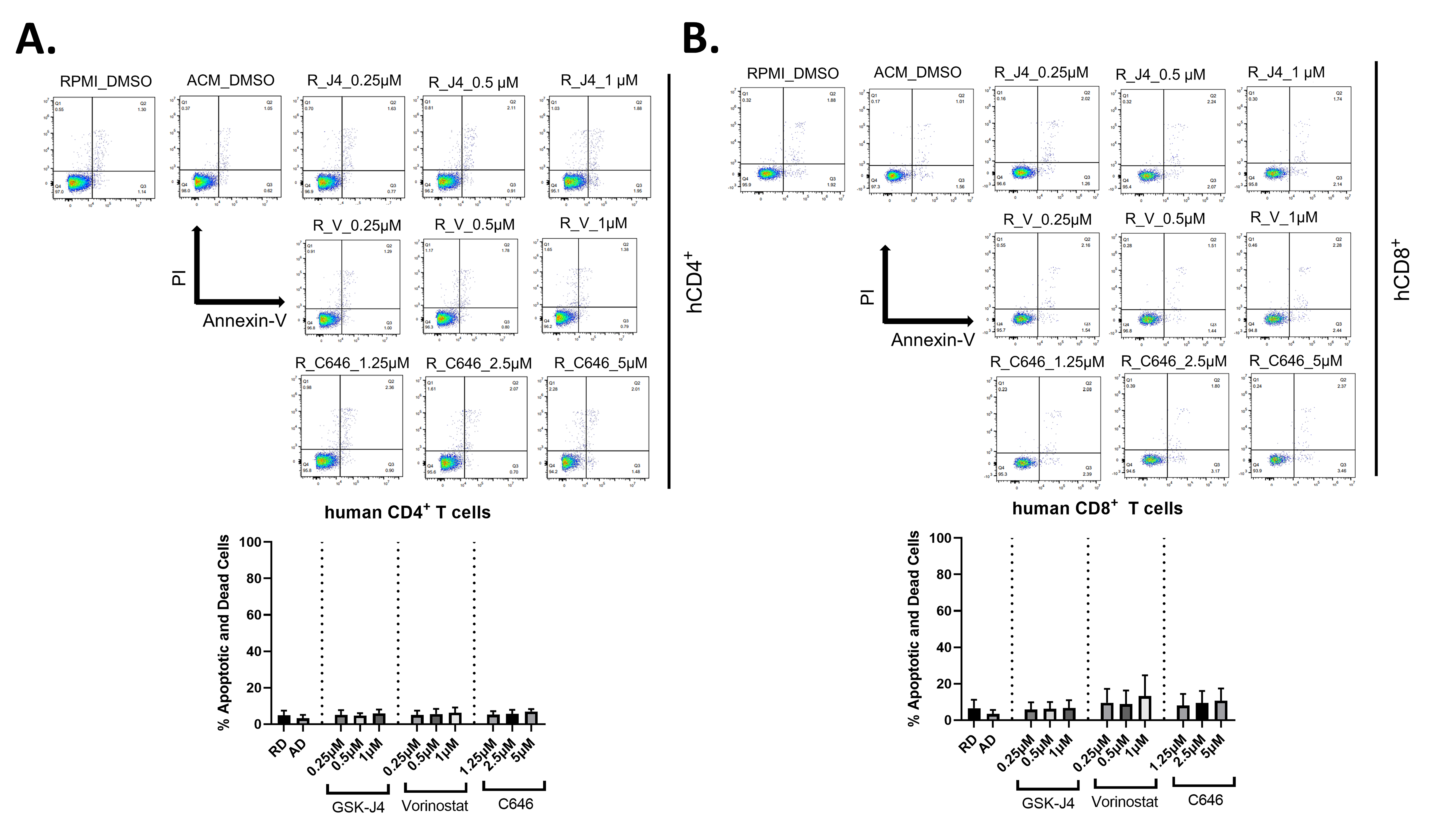

Supplement: Supplementary file 12 [file Image12.tif]
